# Large-Scale Assessment of Mediterranean Marine Protected Areas Effects on Fish Assemblages

**DOI:** 10.1371/journal.pone.0091841

**Published:** 2014-04-16

**Authors:** Paolo Guidetti, Pasquale Baiata, Enric Ballesteros, Antonio Di Franco, Bernat Hereu, Enrique Macpherson, Fiorenza Micheli, Antonio Pais, Pieraugusto Panzalis, Andrew A. Rosenberg, Mikel Zabala, Enric Sala

**Affiliations:** 1 Université de Nice Sophia-Antipolis, Faculté des Sciences, EA 4228 ECOMERS, Nice, France; 2 CoNISMa, Rome, Italy; 3 Centre d'Estudis Avançats de Blanes, CEAB-CSIC, Accés Cala Sant Francesc, Blanes, Spain; 4 Department of Ecology, University of Barcelona, Barcelona, Spain; 5 Hopkins Marine Station, Stanford University, Pacific Grove, California, United States of America; 6 Laboratorio di Acquacoltura e Gestione delle Risorse Acquatiche, Sezione di Scienze Zootecniche, Dipartimento di Agraria, Università di Sassari, Italy; 7 Marine Protected Area of Tavolara-Punta Coda Cavallo, Olbia, Italy; 8 Union of Concerned Scientists, Cambridge, Massachusetts, United States of America; 9 National Geographic Society, Washington, D. C., United States of America; Technical University of Denmark, Denmark

## Abstract

Marine protected areas (MPAs) were acknowledged globally as effective tools to mitigate the threats to oceans caused by fishing. Several studies assessed the effectiveness of individual MPAs in protecting fish assemblages, but regional assessments of multiple MPAs are scarce. Moreover, empirical evidence on the role of MPAs in contrasting the propagation of non-indigenous-species (NIS) and thermophilic species (ThS) is missing. We simultaneously investigated here the role of MPAs in reversing the effects of overfishing and in limiting the spread of NIS and ThS. The Mediterranean Sea was selected as study area as it is a region where 1) MPAs are numerous, 2) fishing has affected species and ecosystems, and 3) the arrival of NIS and the northward expansion of ThS took place. Fish surveys were done in well-enforced no-take MPAs (HP), partially-protected MPAs (IP) and fished areas (F) at 30 locations across the Mediterranean. Significantly higher fish biomass was found in HP compared to IP MPAs and F. Along a recovery trajectory from F to HP MPAs, IP were similar to F, showing that just well enforced MPAs triggers an effective recovery. Within HP MPAs, trophic structure of fish assemblages resembled a top-heavy biomass pyramid. Although the functional structure of fish assemblages was consistent among HP MPAs, species driving the recovery in HP MPAs differed among locations: this suggests that the recovery trajectories in HP MPAs are likely to be functionally similar (i.e., represented by predictable changes in trophic groups, especially fish predators), but the specific composition of the resulting assemblages may depend on local conditions. Our study did not show any effect of MPAs on NIS and ThS. These results may help provide more robust expectations, at proper regional scale, about the effects of new MPAs that may be established in the Mediterranean Sea and other ecoregions worldwide.

## Introduction

Oceans worldwide are threatened by a combination of local direct impacts (e.g. fishing, pollution, sedimentation, coastal development) and diffuse global impacts (i.e. climate change) [1]. These stressors affect not only species and ecological communities [2], but also ecosystem functioning and the capacity of ecosystems to provide essential goods and services to society [3]. In particular, fishing significantly reduces density and biomass of target species; it selectively removes large-sized individuals (locally reducing reproductive potential of stocks), it causes dramatic changes in the structures and functioning of food webs [4], [5], and in the physical properties of seafloor [6], and it may decrease the resilience of populations and ecosystems in the face of climatic impacts and other disturbances [5], [7].

Possible solutions to mitigate the impact of fishing and other human activities on marine ecosystems include the use of spatial management tools such as marine protected areas (MPAs). MPAs can be defined as ‘discrete geographic areas of the sea established by international, national, territorial, tribal or local laws designated to enhance the long-term conservation of natural resources therein’ [8]. MPAs, therefore, are areas where human activities, especially fishing, are restricted or banned [9]. A special case of MPAs is represented by no-take marine reserves where fishing is prohibited. No-take marine reserves have been shown to significantly increase population density, size and biomass of target fishes inside their boundaries [10]. The most effective marine reserves have large fish biomass with a dominance of apex predators, which testifies that they are capable of restoring assemblages to a state close to pristine (see [11] and references therein). In some regions of the world, such as the Mediterranean Sea, MPAs usually include one or more no-take marine reserves surrounded by ‘buffer zones’, where fishing is restricted compared with adjacent fished areas [12]. If effective marine reserves in the Mediterranean have been shown to increase fish biomass [4], [19], [20], the effects of partially-protected MPAs depend on the type of protection and the level of enforcement [14], [21], [22].

In the Mediterranean Sea, fishing has been historically the greatest ecological stressor, depleting target species [13] and altering entire ecosystems [14]. In addition to that, Mediterranean marine ecosystems, especially in coastal areas, have been impacted by the arrival of non-indigenous species and the northward expansion of southern Mediterranean species [15]–[18]. An open question, therefore, concerns to what level MPAs are vulnerable to or may contrast the propagation of Non-Indigenous-Species (NIS) and/or southern thermophilic species (ThS), whose spread is thought to be favored by anthropogenic degradation of marine environments (e.g. from pollution, [23]–[24]) or by the direct effects of a suite of human activities (e.g. navigation and discharge of ballast waters, aquaculture; [25]). Ecological theory (e.g. niche theory; [26]) says that the healthier ecosystems within MPAs could be less favorable to biological invasions by both NIS and ThS. However, the purported function of MPAs in contrasting the spread of NIS and ThS relies just on theoretical ecological bases rather than empirical evidence. In particular, the role of Mediterranean MPAs in limiting the spread of NIS and/or ThS has never been investigated. The potential role of MPAs in limiting or enhancing the spread of NIS and ThS is thus a matter of debate and the available scientific evidence is scarce, unclear and sometimes contradicts the theory. Some study, in fact, showed that invasivability can be positively correlated with biodiversity [26]–[27], while recently Burfeind et al. [28] provided the evidence that the few available studies suggest that marine reserves do not have any limiting effect on or even enhance NIS.

Most of the previous studies conducted worldwide, including in the Mediterranean region, have assessed the responses to protection in one or a few MPAs [10], [19], [22], [29], or across a limited geographical range [30], [31] but see [14], while synthetic studies (e.g., meta-analyses) analyzed published data that are not always consistent in time and methodologies [4], [32], [33]. No study to date has simultaneously examined the role of MPAs in reversing the effects of overfishing and in possibly limiting the spread of NIS and/or ThS over large spatial scale in the Mediterranean. Sala et al. [14] examined variation in whole rocky reef community structure across the Mediterranean basin, but the specific question about how MPAs may promote recovery of fish assemblages from fishing remains. Moreover, Sala et al. [14] did not examine the possible role of MPAs in limiting the spread of NIS and ThS. Finally, most studies on MPAs' effects to date have contrasted MPAs with adjacent fished areas. This approach cannot be used over large scales because of logistical and technical constraints. Thus, empirical evidences of the general effects of MPAs in recovering fish communities and maintaining native diversity across large scales are still lacking, and this is the case of the Mediterranean Sea.

In this study, we investigate the responses to protection of fish assemblages associated with shallow rocky reefs from MPAs and areas open to fishing, across a wide geographical gradient across the Mediterranean Sea (from Spain to Turkey). Because controlled experiments testing the large-scale effects of human impacts or multiple stressors (e.g. fishing and climate change) are impractical, we compared communities at sites distributed along gradients of human disturbance to examine community change across these gradients using a “space-for-time” approach [2], [34].

Specifically, we addressed the questions: (1) do fish communities respond coherently to protection over a regional scale (1000 s km)? (2) do the same species drive the trajectory of functional recovery in no-take zones at regional scale? (3) have MPAs any effect on the spread of NIS and ThS fishes in the Mediterranean Sea?

## Materials and Methods

### Sampling area and methods

**Figure 1:**
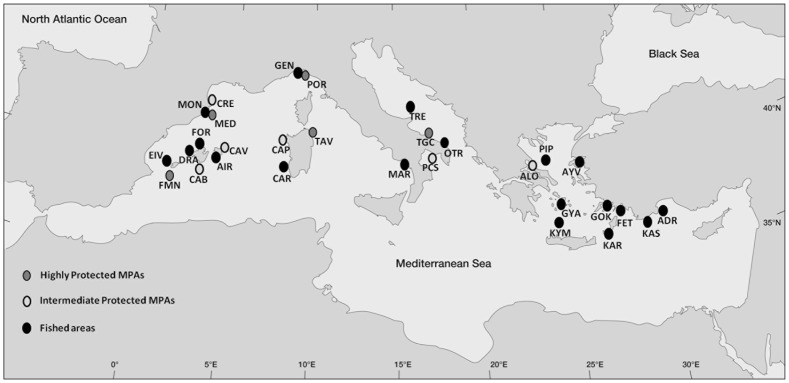
Map of study sites classified basing on protection categories. ADR = Adrasan, AIR = Illa de l'Aire, ALO = Alonissos, ALP = Al-Hoceima MPA, AYV = Ayvalik, CAB = Cabrera, CAP = Capo Caccia, CAR = Carloforte, CAV = Cap de Cavalleria, CRE = Cap de Creus, DRA = Dragonera, EIV = Eivissa, FET = Fethiye, FMN = Formentera-Espardell, FOR = Cap Formentor, GEN = Genoa, GOK = Gokova, GYA = Gyaros, KAR = Karpathos, KAS = Kas, KIM = Kimolos-Polyaigos, MAR = Maratea, MED = Medes Islands, MON = Montgrí, OTR = Otranto, PCS = Porto Cesareo, PIP = Piperi, POR = Portofino, TAV = Tavolara, TGC = Torre Guaceto, TRE = Tremiti.

Fish assemblages were surveyed at 13 marine protected areas and 17 unprotected areas located across the northern Mediterranean Sea in May–June 2007 and 2008 (Fig. 1, Table S1).

The following institutions provided research permits: Medes Islands Marine Reserve, Cap de Creus Natural Park, Marine Resources Service and Biodiversity Department of the Balearic Islands Government, Archipelago of Cabrera National Park, Al Hoceima National Park, Portofino National Marine Reserve, Torre Guaceto Marine Protected Area, Tavolara-Punta Coda Cavallo Marine Protected Area, Porto Cesareo Marine Protected Area, Tremiti Marine Reserve, Capo Caccia Marine Protected Area, National Marine Park of Alonissos, Northern Sporades, and the Ministry of Foreign Affairs of Turkey.

We surveyed rocky reefs at 8–12 m depth, at 4–6 replicate stations within each area, depending on their extension. Sampling stations within areas were separated at least 1 km apart from the next, except in very small marine reserves (e.g., Portofino, Piperi) where stations were sampled hundreds of meters away from each other in order to have enough replicate surveys.

Fish assemblages were sampled underwater using visual census along 3 replicate instantaneous strip transects of 25×5 m [35] at each station. Visual censuses were performed on rocky substrates where other substrate types, like sand or seagrasses, represented less than 5% in cover (both within and around transects). Along each transect, the diver swam one way at constant speed (approximately 4 meters/min.), identifying and recording the number and size of each fish encountered. Fish size (total length: TL) was recorded within 2-cm size classes for most of the species, and within 5-cm size classes for large-sized species such as *Epinephelus marginatus*. Fish wet mass was estimated from size data by means of length-weight relationships from the available literature [36], [37].

The assessment of protection effects on fish assemblages can be influenced by habitat complexity [38]. Substrate rugosity was measured *in situ* for each replicate transect, at all stations (both protected and unprotected), to account for variability in fish assemblages due to rugosity as a covariate. To measure rugosity, a 10-m long small-link chain (1.3 cm per link) was draped along the length of the centerline of each transect. Care was taken to ensure that the chain followed the contour of all natural fixed surfaces directly below the transect centerline. A ratio of 10 to linear horizontal distance between the beginning of the transect and the end of the draped chain gave an index of rugosity (see [14]).

We focused on variability in habitat complexity (i.e. rugosity) at a spatial scale that, from literature available, was shown to be relevant in structuring Mediterranean fish assemblages (see [38] for detailed discussion) and that matches the spatial scale of the sampling unit adopted in the present study (i.e. 25*5 m transect).

To measure the level of protection, data from each area were pooled into the following three categories, in decreasing order of protection: (a) well-enforced no-take MPAs (HP: Highly Protected) (Formentera-Espardell, Medes, Portofino, Torre Guaceto, Tavolara), (b) MPAs where some fishing is allowed or some illegal fishing may occur due to weak enforcement (IP: Intermediate Protection) (Cabrera, Cap de Creus, Capo Caccia, Porto Cesareo, Cavalleria, Alonissos), (c) non-enforced MPAs (Piperi, Tremiti) plus 17 open access areas (F: Fished). MPAs were categorized using information from the scientific literature, personal experience, and interviews with MPAs' staff reporting on the overall management effectiveness (see [14]).

Unprotected areas are typically open-access with little enforcement of fishing regulations. To minimize differences possibly deriving from other human threats combined to fishing, sites were selected within areas not directly affected by evident sources of impact (e.g. harbors, defense structures, sewage outfalls, extensive urbanization).

Species were classified into three commercial categories: commercial species (C), species with low commercial value (LC), and species with null commercial value (NC), according to [33] and to [37] (see Table S2). Species were also classified in trophic groups according to [14], (Table S2). Non-indigenous species (NIS) were identified according to [17], while thermophilic species (ThS) were identified according to [39].

### Data analyses

The effects of different protection regimes on overall fish assemblage structure (i.e. taxonomic composition and relative contributions of fish taxa in terms of density or biomass) were analyzed using multivariate statistical techniques. Specifically, taxon x sample matrices (n = 82 taxa, n = 514 samples) were analyzed using unbalanced three-way permutational multivariate analysis of variance (PERMANOVA, Anderson 2001). ‘Protection’ (Pr) was considered as a fixed factor (3 levels, as classified above), ‘Site’ (Si) was a random factor (up to 19 levels) nested in Pr, and ‘Station’ (St) was a random factor (up to 6 levels) nested in Si.

The effects of different protection regimes on relevant fish community variables (i.e. taxon richness; total fish density and biomass; density and biomass of each commercial and trophic group) were analyzed using univariate techniques. Specifically, univariate PERMANOVA based on Euclidean distance measure [40] was used in order to avoid any assumption about the distribution of the variables [41]. In this analysis P-values associated with F statistics are obtained by permutation. Rugosity was a covariate in all analyses. For NIS and ThS, latitude and longitude were also included as covariates. Pairwise tests were run whenever appropriate. Potential difference in rugosity among different protection levels was tested using an univariate PERMANOVA based on Euclidean distance measure following the experimental design described above.

Density and biomass of different trophic levels were plotted in trophic pyramids considering, for the sake of clarity, only two levels of protection, HP and F+IP, with these latter pooled together due to the general absence of significant differences (see Results).

In order to assess potential patterns of taxon richness along both latitudinal and longitudinal gradients, we used polynomial regressions with a parabolic shaped relation [42].

To explore whether the same taxa contributed to the response to protection over the regional scale, across different MPAs, PERMANOVA was also carried out on the 5 HP MPAs considering ‘Site’ (Si; 5 levels) and ‘Station’ (St; up to 6 levels) as random factors (up to 6 levels) nested in Si. To visualize multivariate patterns, non-metric multidimensional scaling (nMDS) ordinations were obtained from Bray-Curtis dissimilarity matrices where only the 5 centroids for the factor Site were visualized. Stress values were shown for each MDS plot to indicate the goodness of representation.

The PRIMER 6 and Permanova+B20 package (Plymouth Marine Laboratory) was used to perform the analyses [43]. Polynomial regression was performed using the open source software ‘R’ (see www.r-project.org).

## Results

No difference in rugosity was highlighted among different levels of protection (pseudo-f = 0.21, p = 0.80, Fig. S1), while significant variability was recorded both at the scale of sites (pseudo-f = 6.40, p<0.001) and stations (pseudo-f = 1.66, p p<0.001).

The level of protection had clear effects on the composition and structure of rocky reef fish assemblages across the Mediterranean Sea.

**Figure 2:**
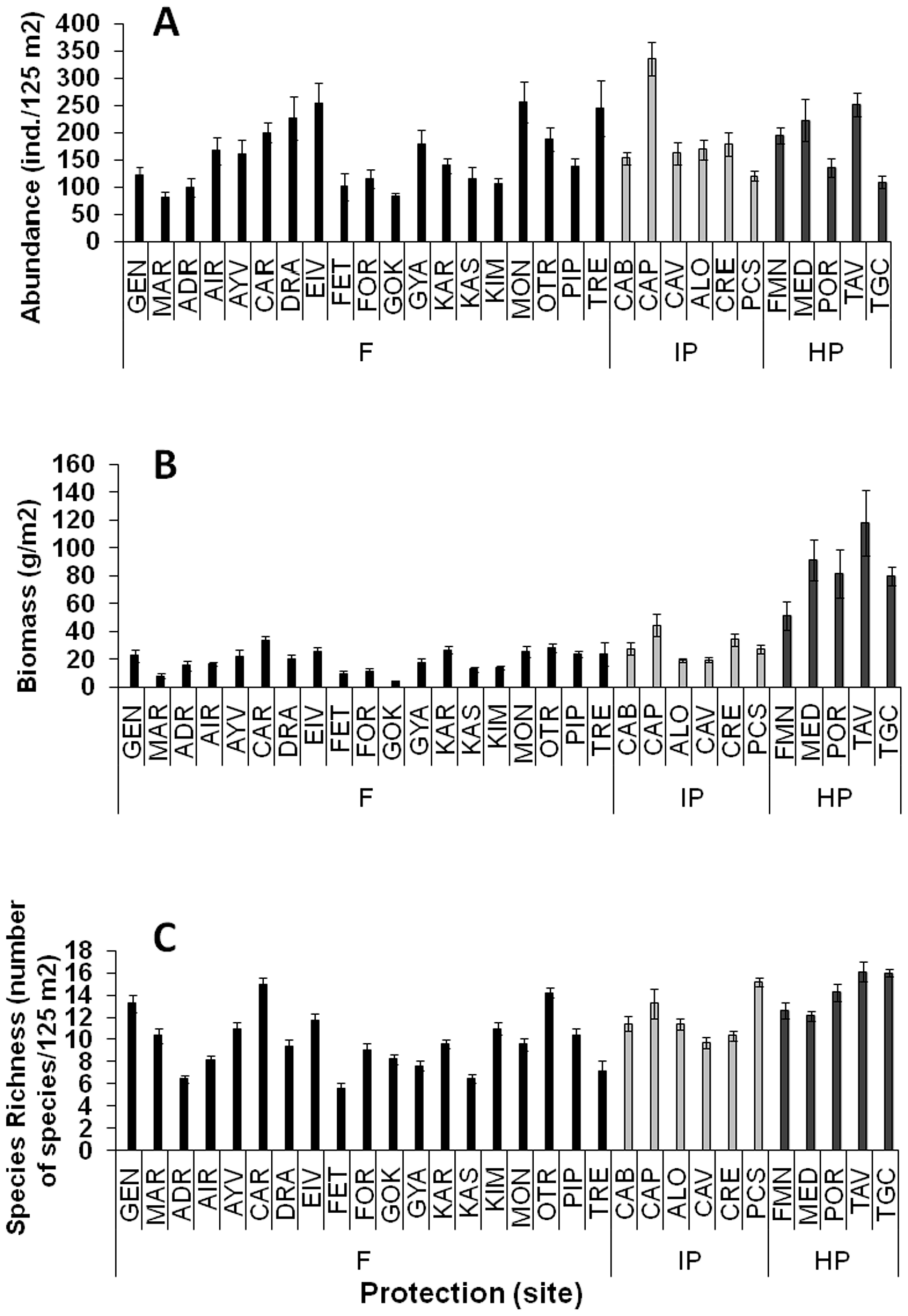
Mean values (± SE) of a) total fish density, b) total fish biomass, and c) fish species richness at each sampling site (see Figure 1 for complete site names). Black bars indicate fished areas (F), light gray bars indicate intermediate protected MPAs (IP) and dark gray bars indicate highly protected MPAs (HP).

Total density of fish (all taxa pooled) did not differ among protection levels (Table 1, Table S3a, mean±SE: 163.71±4.82 ind.*125 m^−2^), although it varied significantly among stations and sites (Fig. 2A). Density increased significantly with increasing rugosity following a linear relationship (DISTLM, n = 513, pseudo-f = 12.97, p<0.01).

**Table 1:** PERMANOVA summaries for factor Protection on square root transformed univariate data.

Univariate analyses	Spp Richness		Density		Biomass	
	pseudo-F	p	pseudo-F	p	pseudo-F	p
**All Species**	6.1535	0.006	0.94722	0.3989	40.567	0.0001
**Apex Predators**	NA		25.045	0.0001	37.331	0.0001
**Carnivores**	NA		5.3281	0.0111	18.692	0.0001
**Planktivores**	NA		0.24833	0.7885	2.5751	0.097
**Herbivores**	NA		9.627E-2	0.9091	0.16482	0.8503
**Detritivores**	NA		4.8952	0.018	6.0062	0.0062
**NC**	NA		0.17406	0.8443	0.93531	0.4069
**LC**	NA		1.0548	0.3608	5.5419	0.0097
**C**	NA		13.319	0.0001	77.166	0.0001

NC = species with null commercial value, LC = species with low commercial value, C = species with high commercial value (see material and methods section for details).

Total biomass of fish was greatest at HP, followed by IP and then F (Table 1, Table S3b, mean: 83.4±6.9, 29.8±2.0, 10.0±0.8 g*m^−2^; Fig. 2B, pairwise tests: HP>IP>F; p<0.01). Like density, biomass was significantly variable at the scale of station and site, and was positively linearly-related with rugosity (DISTLM, n = 513, pseudo-f = 7.96, p<0.01).

**Figure 3:**
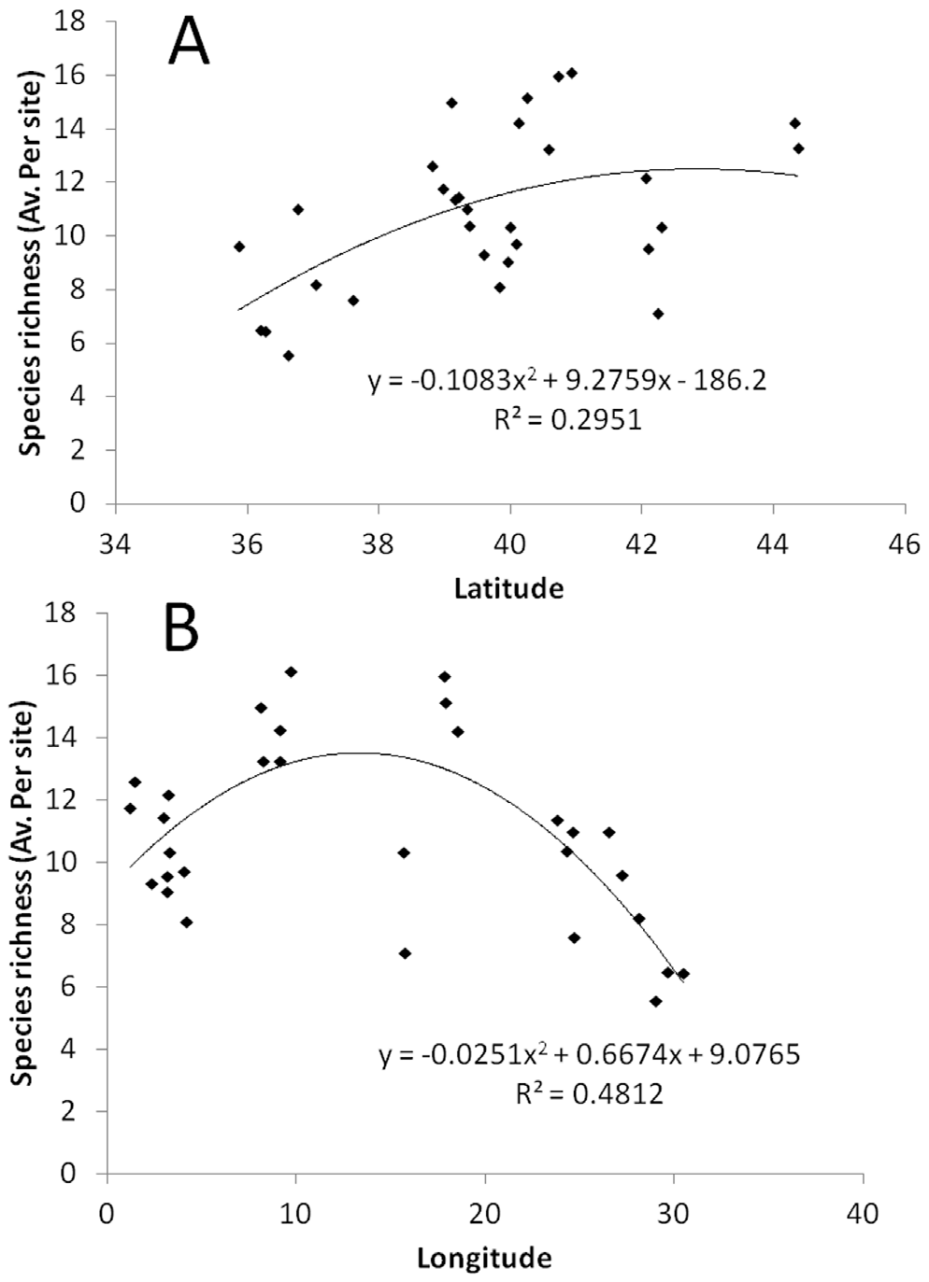
Patterns of average species richness per site versus a) latitude and b) longitude. Lines indicate 2^nd^ order polynomial regression fitting.

Species richness was significantly greater at HP compared to F, while no significant differences were observed between IP and the other protection levels (Table 1, Table S3c, mean: 14.2±0.3, 11.9±0.4, 9.8±0.2 taxa*125 m^−2^ at HP, IP and F, respectively; Fig. 2C, pairwise tests: HP>F, HP = IP, IP = F; p<0.01). Species richness was significantly variable at spatial scales of station and site but did not vary with rugosity. Species richness increased with increasing latitude following a parabolic-shaped curve (Fig. 3A; p<0.01 for 2^nd^ order polynomial regression test). The relationship between richness and longitude was hump-shaped (p<0.001 for 2^nd^ order polynomial regression test), with highest species richness recorded between 10–20°E of longitude (Fig. 3B).

**Figure 4:**
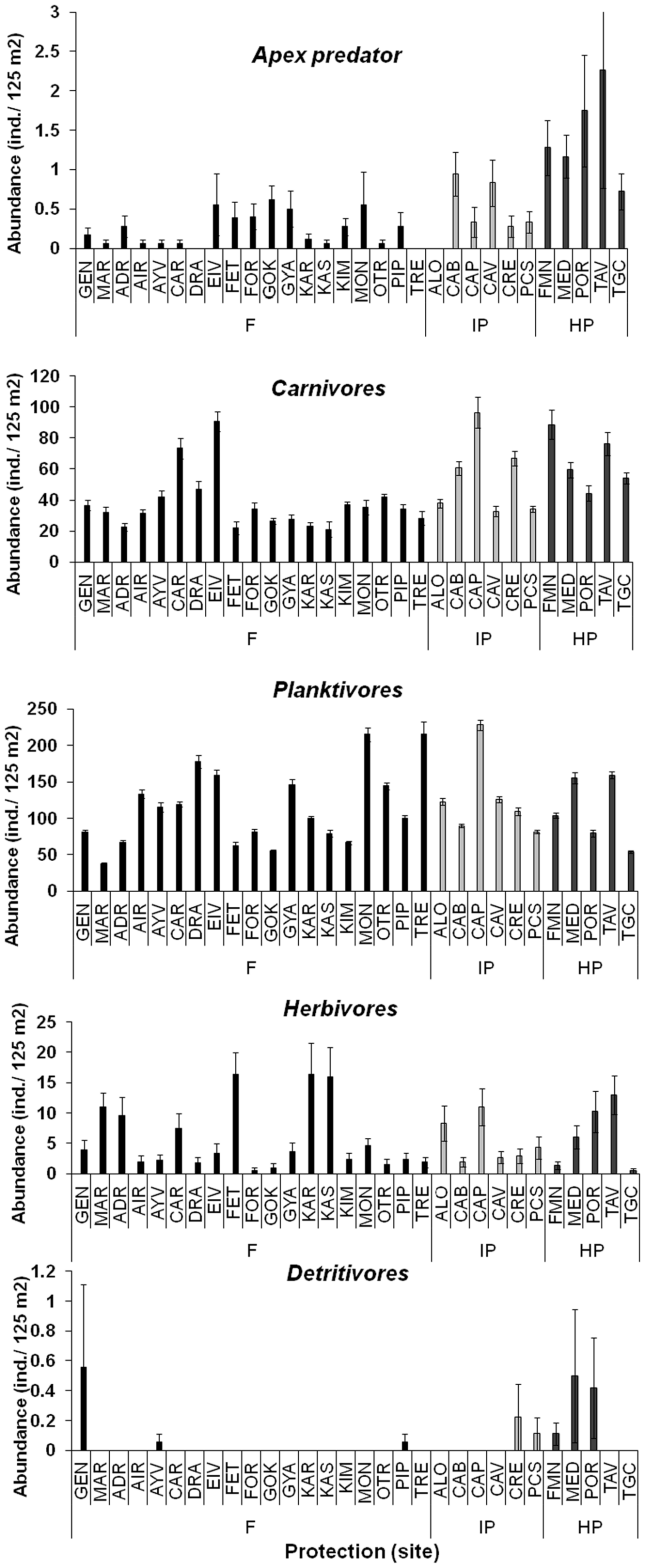
Mean values (± SE) of density per transect per each trophic group at each sampling site (see Figure 1 for complete site names). Black bars indicate fished areas (F), light gray bars indicate intermediate protected MPAs (IP) and dark gray bars indicate highly protected MPAs (HP).

**Figure 5:**
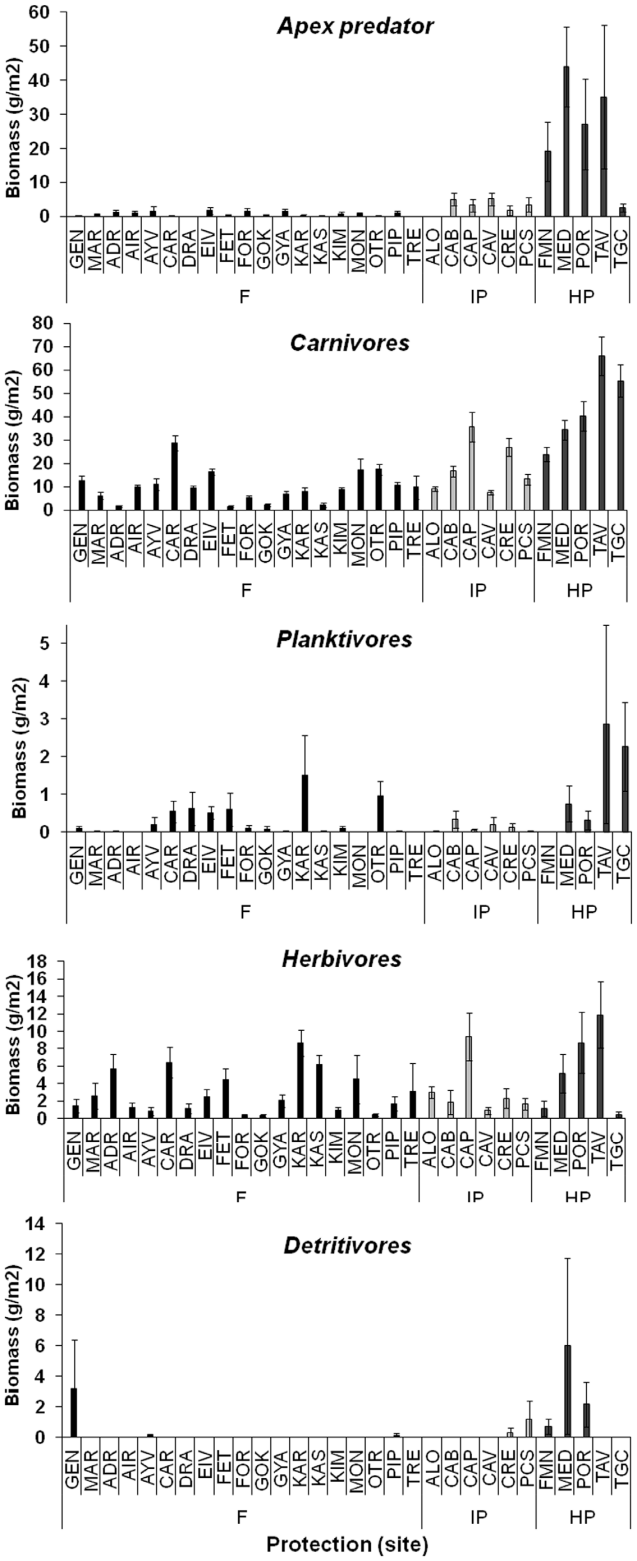
Mean values (± SE) of biomass per transect per each trophic group at each sampling site (see Figure 1 for complete site names). Black bars indicate fished areas (F), light gray bars indicate intermediate protected MPAs (IP) and dark gray bars indicate highly protected MPAs (HP).

The different trophic groups varied in their responses to protection, with significant positive effects on apex predators, carnivores and detritivores, but not on planktivores and herbivores (Table 1, Table S3d–S3m). Total density and biomass of apex predators were significantly greater at HP (1.4±0.3 ind.* 125 m^−2^ and 25.1±5.6 g*m^−2^), followed by IP (0.5±0.1 ind*125 m^−2^ and 3.1±0.8 g*m^−2^) and F (0.2±0.1 ind.*125 m^−2^ and 0.7±0.1 g*m^−2^; Fig. 4, 5). The same pattern was observed for carnivores: HP (65.7±3.3 ind.*125 m^−2^ and 43.4±3.0 g*m^−2^)>IP (52.3±3.0 ind.* 125 m^−2^ and 17.2±1.6 g*m^−2^)>F (37.5±1.3 ind.*125 m^−2^ and 9.8±0.6 g*m^−2^; HP>IP>F; Fig. 4, 5). Total density and biomass of detritivorous fishes were significantly greater at HP than at F (for total density mean±S.E.: 0.19±0.11, 0.05±0.04, 0.03±0.03 ind.*125 m^−2^ at HP, IP and F, respectively; for total biomass 1.80±1.30, 0.26±0.21, 0.18±0.17 g*m^−2^ at HP, IP and F, respectively; in both cases pairwise tests: HP>F, HP = IP, IP = F; p<0.01) (Table 1; Fig. 4, 5). Density and biomass of planktivorous and herbivorous fishes did not show any difference among protection levels (Table 1; Fig. 4, 5).

**Figure 6:**
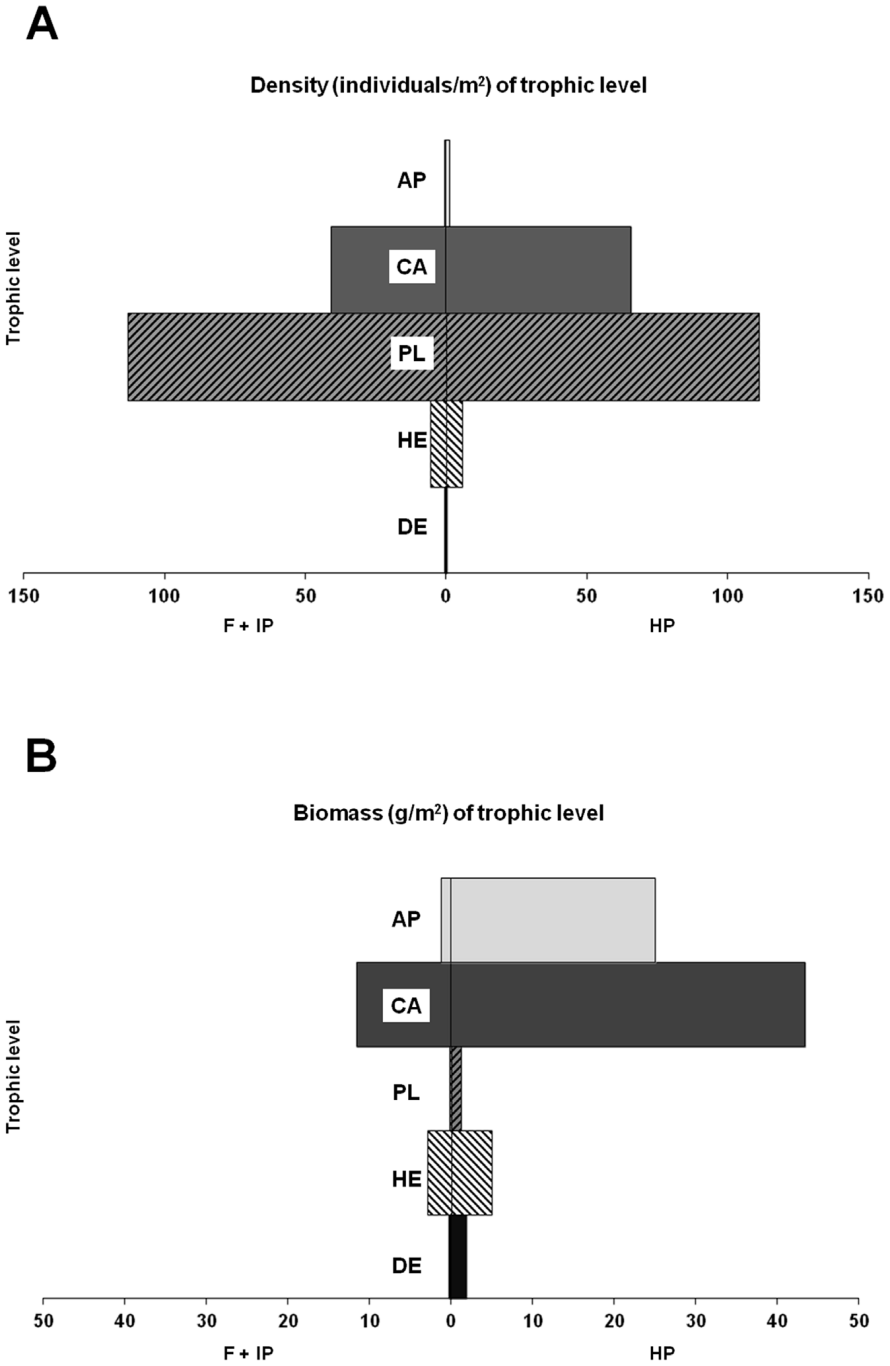
Trophic “pyramids” expressed in term of densities (A) and biomasses (B) for each trophic level. Only two levels of protection were considered (high protection vs weak protection+fished). PL = planktivore, DE = detritivore, CA = carnivorous, AP = apex predator, HE = herbivorous.

When density and biomass of different trophic levels in trophic pyramids was examined (Fig. 6), significant differences emerged between HP and F+IP in terms of biomass (p<0.01, chi squared test), while differences were not significant in terms of density (p<0.05, chi squared test). Planktivores numerically dominated fish assemblages, whatever the protection level. In terms of density, all trophic groups were equivalent between HP and F+IP conditions, except for carnivores and apex predators that tended to be more abundant in HP conditions, although differences were not significant (Fig. 6A). Differences between HP and IP+F in terms of biomass were more pronounced and statistically significant: all trophic groups showed greater biomass in HP than IP+F, particularly the apex predators and carnivores (Fig. 6B).

Considering density data, assemblage structure of commercial fishes did not differ among protection levels (Table 2, Table S4a). Total density of commercial species (all species pooled) was significantly greater at HP and IP than in F (Table 1; Table S3n; pair-wise tests: HP = IP>F; p<0.05). Considering biomass data, the assemblage structure of commercial fishes was significantly different between HP and F (Table 2; Table S4b; pairwise tests; p<0.05). Total biomass of commercial species (all species pooled) was greater at HP than at IP and F (Table 1; Table S3o; mean: 57.1±6.5, 13.3±1 and 5.6±0.4 g*m^−2^ in HP, IP and F respectively; pairwise tests: H>IP>F; p<0.01).

**Table 2:** PERMANOVA summaries for factor Protection on square root transformed multivariate data.

Multivariate analyses	Density		Biomass	
	pseudo-F	p	pseudo-F	p
**All Species**	2.1458	0.0065	2.8684	0.0003
**Apex Predators**	11.04	0.0001	12.181	0.0001
**NC**	2.1154	0.0483	2.272	0.0307
**LC**	1.6089	0.1303	1.8485	0.0856
**C**	1.7555	0.0716	2.9418	0.0011

NC = species with null commercial value, LC = species with low commercial value, C = species with high commercial value (see material and methods section for details).

Assemblage structures (both considering density and biomass data) and pooled densities of low-value fishes did not differ among protection levels (Table 1, 2, Table S3p, S4c,d), while significant differences were recorded at the scales of station and site. Total biomass of low-value species (all species biomasses pooled) was greatest at HP, intermediate at IP and lowest at F (mean: 83.4±6.8, 29.7±2.1 and 18.9±0.7 g*m^−2^ in HP, F and IP, respectively; pairwise tests: HP>IP>F; p<0.05, Table 1, Table S3q).

Assemblage structures of fish of null commercial value (based on density data) significantly differed among protection levels (Table 2, Table S4e) only between HP and F (pairwise tests: HP>F, HP = IP, IP = F), while no significant difference was detected in terms of total density (all species density data pooled; Table 1, Table S3r).

Assemblage structures based on biomass data of fish of null commercial value significantly differed among protection levels (Table 2, Table S4f) with significant difference only between HP and F (pairwise tests: HP>F, HP = IP, IP = F), while total biomasses (all species biomasses pooled) did not display any pattern related with protection levels (Table 1, Table S3s).

**Figure 7:**
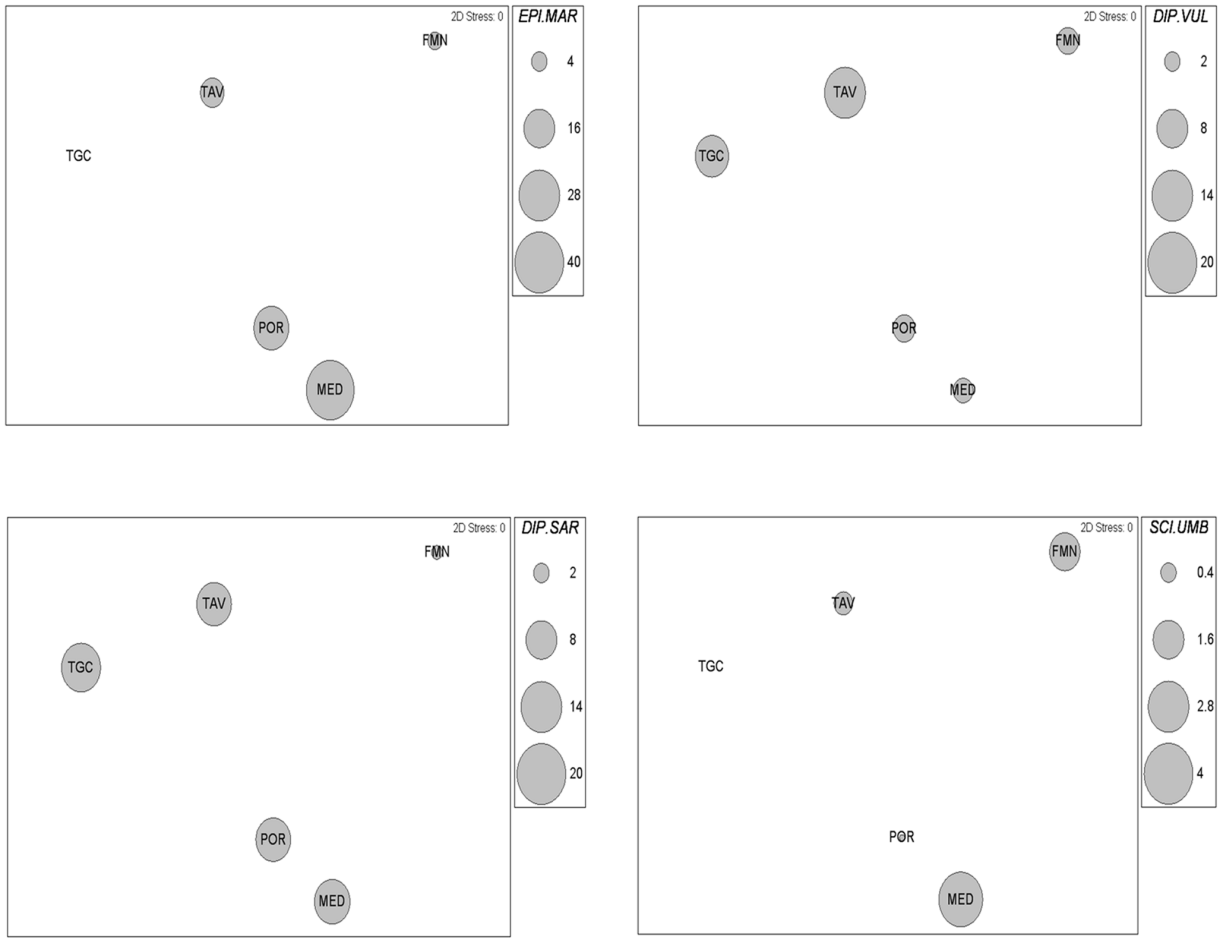
Fish assemblage structures based on biomass data. Two-dimensional nMDS ordinations of centroids of the 5 MPAs classified as HP are shown. Bubble values indicate average biomass of a subset of fish species responding to protection.

To explore the generality in commercially valuable fish responses to protection at large scales, biomass of these species were analyzed using multivariate analysis. A significant variability was detected among the five HP MPAs (pseudo-f = 3.70, p<0.0001). The dusky grouper *Epinephelus marginatus* showed the highest biomass at Medes Islands followed by Portofino and to a lesser extent Tavolara. The two-banded sea bream *Diplodus vulgaris* displayed the highest biomass at Tavolara MPA followed by Torre Guaceto MPA, while white seabream *Diplodus sargus* showed similar values at all four MPAs except Formentera. Finally, the highest biomass of brown meagre *Sciaena umbra* was found at Medes, followed by Formentera and to a lesser extent Tavolara. Dusky grouper and brown meagre were absent from Torre Guaceto (Fig. 7).

**Figure 8:**
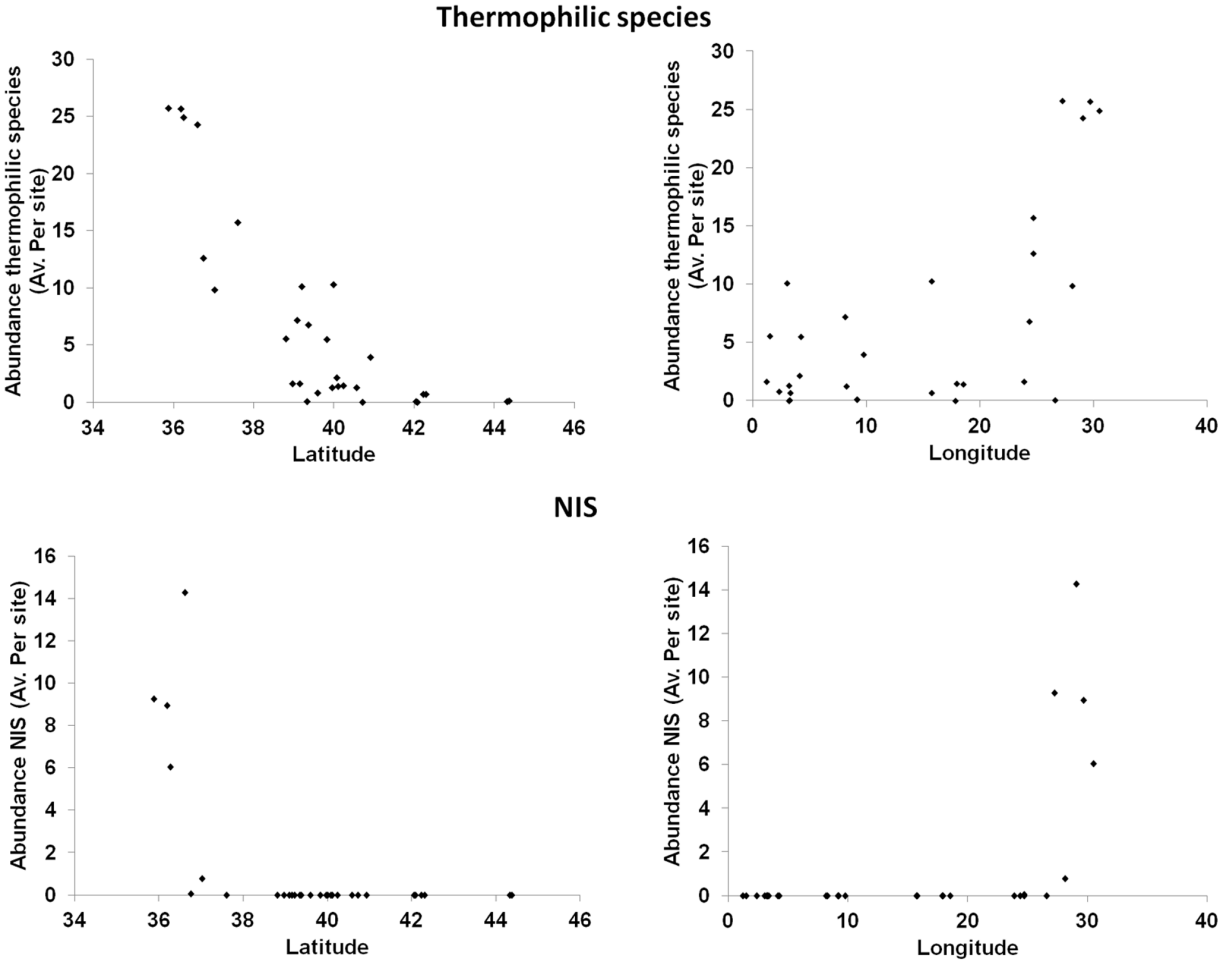
Patterns of average density of thermophilic and NIS species per site versus latitude and longitude.

Eleven ThS were censused, some of them being also NIS: *Fistularia commersoni*, *Pteragogus pelycus*, *Sargocentron rubrum*, *Siganus luridus*, *Siganus rivulatus*, *Pomatomus saltatrix*, *Thalassoma pavo*, *Sparisoma cretense*, *Epinephelus caninus*, *Epinephelus costae* and *Sphyraena viridensis*. Assemblages of ThS (in terms of multivariate densities and total density i.e. all species density pooled), did not respond to protection (for multivariate densities pseudo-f = 1.21, p>0.2; for total densities pseudo-f = 0.15, p>0.9). Total density of ThS decreased with increasing latitude (Fig. 8; Linear regression analyses, DISTLM, n = 30, pseudo-f = 51.18, p<0.001) and was significantly and positively related to rugosity (linear regression analyses, DISTLM, n = 513, pseudo-f = 4.79, p<0.05).

Five NIS were censused in this study: *Fistularia commersoni*, *Pteragogus pelycus*, *Sargocentron rubrum*, *Siganus luridus* and *Siganus rivulatus*. All NIS censused were also ThS (representing in fact a subset of all ThS censused). NIS assemblage structures and their total density (i.e. all species density pooled) did not show any response to protection, longitude and rugosity. A significant relation with latitude, on the contrary, was found (PERMANOVA, pseudo-f = 18.24, p<0.001): total density of NIS fish suddenly decreases with increasing latitude, dropping to zero above at coordinates approximately corresponding to 38°N and 25°E (Fig. 8), i.e. at the line connecting southern Balearic Islands to southern Tyrrhenian Sea, and at the Aegean Sea.

## Discussion

Our region-wide survey of Mediterranean rocky reef fish assemblages clearly shows significantly higher fish biomass in no-take MPAs relative to partially-protected MPAs and open access fishing areas. Partially-protected MPAs are closer to open access fishing areas along the recovery trajectory from unprotected areas to HP MPAs. These findings are generally consistent with the evidence arising from previous field studies dealing with single MPAs [22], [44] or meta-analyses using data from multiple MPAs [4], [21], [33]. This is, however, the first field study showing MPA effects on fish at this large scale and using consistent methods and design. Thus these findings obtained in a field study highlight the generality of the effects of protection on reef fish assemblages, over spatial scales not addressed before. Importantly, we found that a high degree of protection (no or minimal fishing) always resulted in increased fish biomass and in the density of carnivores and apex predators. Previous idiosyncratic results of studies from MPAs are likely due to variable levels of protection of the focal locations.

Greater biomass in HP MPAs is driven by a positive response of fishes with commercial value (either high or low) [4], [33], [38], [44], but no significant differences were found for fish with null commercial value. This result further supports our conclusion that fishing is a major driver of the density and structure of these assemblages. Non-commercial species are expected not to be directly affected by protection, and any response to protection can be ascribed to indirect effects, e.g. via food web interactions [45]. The absence of any protection effect on fishes with null commercial value in our dataset could be due to the weakness of trophic interactions specifically involving piscivorous fishes and their fish prey [46]–[47], possibly because large-sized piscivorous predators (e.g. seals, sharks, groupers, common dentex) are at low levels, relative to historical populations, along most Mediterranean coastal habitats [13], [14], [48], [49].

High trophic levels (i.e. apex predators, carnivores) showed both higher density and biomass within HP MPAs than to in IP MPAs and fished areas. On the contrary, herbivorous and planktivorous fishes did not display any response related to protection from fishing, as previously highlighted by Guidetti and Sala [4]. As a consequence, within HP MPAs the trophic structure of fish assemblages resembles a top-heavy (i.e. inverted) biomass pyramid [50], a pattern that has been reported from remote unfished sites (e.g. [5], [51], [52]), with most cascading effects occurring via benthic community components (e.g. sea urchins and erect algae; [53]). From this perspective, a recent paper by Trebilco et al. [50] indicates that biomass pyramids are usually expected to be bottom-heavy for communities that share a common resource base and the authors state that top-heavy biomass pyramids can arise from visual census artifacts or energetic subsidies. Sampling artifacts can arise from the adoption of non-instantaneous underwater visual census (UVC) techniques that can overestimate density and biomass of large piscivorous fishes (see [54] for a detailed discussion). This bias can occur especially in presence of predator fishes displaying high swimming speed and attractive behavior towards divers. In our study we can reasonably exclude a significant sampling bias because 1) we adopted an instantaneous visual census approach and 2) predators in the analyzed ecosystem are not particularly fast-swimming species that can be attracted by divers. Sharks were absent in our sampling sites [14] and groupers were the largest predators along with other piscivorous fishes (e.g. *Dentex dentex*, *Seriola dumerili*). From this perspective, we can hypothesize that top-heavy biomass pyramids we found in HP MPAs are cases of subsidized ecosystem compartments, where large predators have access to more production than do smaller members of the community (i.e. mobile consumers access production from multiple local biomass pyramids, hence escaping the constraints of energy availability at local scales, [50]).

In this study we highlighted a significant effect of habitat rugosity on some variables possibly related to MPA effectiveness (i.e. total fish density, total fish biomass). Particularly, transects (i.e. 125 m^2^ areas) with high rugosity supported fish assemblages characterized by higher density and biomass. A significant positive effect of rugosity was also recorded on the density of ThS. These evidences are in agreement with the previously recognized importance of habitat structure in affecting fish assemblages [38], [55]. On the other hand, we did not highlight any potential confounding effect among protection and habitat complexity (as suggested in [38], [55]) because of the absence of significant differences in rugosity among sites at different protection levels. In our study there was no evidence that Mediterranean MPAs are established in zones harbouring particularly structurally complex habitats, at least at the scale investigated.

Although the functional structure of the fish assemblages was consistent among HP MPAs, the species accounting for the differences between HP MPAs, and IP MPAs and open access areas differed among locations. For example, in Torre Guaceto, the sea breams *Diplodus sargus* and *D. vulgaris* determined the response to protection, whereas other species that are classically related with the reserve effect, like the dusky grouper *Epinephelus marginatus*, were absent. The dusky grouper, conversely, contributed more than the other species to determine the response to protection at the Medes Islands (along with *Sciaena umbra*), Portofino (with *D. sargus*) and Tavolara (with all three other species). *Sciaena umbra* contributed considerably to differentiate protected assemblages at Formentera (together with *D. vulgaris*) and Medes (together with *E. marginatus*). These differences are probably related to local environmental conditions (e.g., availability of habitat types, habitat complexity and heterogeneity, depth, slope, and temperature) [56]. This suggests that the recovery trajectories in HP MPAs are likely to be functionally similar (i.e. represented by predictable changes in trophic groups), but the composition of the resulting assemblages may depend on local environmental conditions.

In the present study we did not investigated the effect of MPAs age (i.e. time in years since the inception of protection) on MPAs effectiveness because our sampling design did not include temporal replication in each MPA. Simply testing for potential correlation among years of protection and some relevant variables (e.g. total fish biomass, biomass of apex predator, biomass of commercially valuable fishes) we would have included a spatio-temporal confounding in the test for MPA effectiveness (i.e. different years since protection would correspond to MPAs located in different areas). This point is particularly relevant especially considering the variability in recovery patterns we highlighted among different effective MPAs. In order to properly evaluate the effect of time elapsed from the inception of protection on fish assemblages, long time series are needed for each MPA. Unfortunately, just in a few cases such long time series are available (e.g. [57] for the case of a Mediterranean MPA) and major effort should be done to fill this gap.

However, it is valuable to acknowledge that all the HP MPAs we considered were implemented at least 9 years before our survey (i.e. Formentera, Spain), this time exceeding the stint previously estimated for an MPA to become effective (see [33], [44], despite for some species much longer period can be needed to fully recover, [57]).

Results of our large-scale survey also show that the general pattern of species richness of coastal fishes does not follow an eastward decline, as assumed by Quignard and Tomasini [58]. Our data reveal highest species richness at 10°–20°E longitude. This pattern matches the evidence reported by [42] concerning a pool of 619 Mediterranean fish taxa associated to a wide array of habitats (e.g. rocks, sand, seagrasses) in coastal waters but also in the open seas. Our results suggest that coastal fish species richness could be used as a proxy for describing patterns of fish species richness at regional scale in the Mediterranean Sea.

At the local scale of individual areas, species richness was significantly greater within HP Mediterranean MPAs. Controversial evidences are available from the global literature on the expected effects of MPAs on diversity, with some studies revealing an effect of protection on species richness and other studies failing to detect any significant difference (see [8] for a detailed discussion about possible causes). Our results indicate that protection has general positive effects on species richness of reef-associated fish assemblages.

With regard to the possible effects of MPAs in contrasting the spread of NIS, although at small spatial scales biodiversity may confers invasion resistance, at larger scales biodiversity seems to have no effects or even enhance number and densities of NIS [26], [59]–[61]. A number of putative mechanisms have been invoked to explain the enhancement of NIS invasions in places characterized by high species richness, such as a greater habitat heterogeneity that could favor the settlement and propagation of NIS (see e.g. [27]). Additionally, several human-mediated ecological processes can facilitate the establishment of a NIS inside a marine reserve, such as the prevention of their harvest or lower competition from native species by increasing their predators and parasites [28]. On the other hand, there are also putative mechanisms that limit the success of NIS within MPAs, such as an increased competition for space and other resources, as well as a stronger top-down regulation [23], [24], [62], but until now this remains a theoretical framework not yet supported by empirical studies.

Our study did not show any effect of MPAs on NIS nor Mediterranean ThS fishes, which showed comparable densities between MPAs and unprotected areas. Therefore, the greater species diversity of fish we documented in MPAs does not appear to result in lower invasibility. The lack of observable effects of MPAs on NIS fish densities suggests that the mechanisms of invasion are not affected by protection.

Fish NIS distribution was restricted to the areas located south of an imaginary line connecting the Baleares Islands to the southern Tyrrhenian Sea. This finding, based on field sampling at a basin scale, mostly agrees with the patterns obtained on the basis of literature review [63] and further supports the idea that the Mediterranean Sea is under invasion of NIS fishes (most species being actually thermophilic) that mostly entered the Mediterranean via the Suez Canal [64]. From this perspective, thermophilic NIS could have benefited from global warming by expanding their ranges northwards [15]. Dispersal rate of marine species in response to climate change is estimated on average to be about 19 km yr^−1^ and it is generally assumed that NIS are better dispersers compared to native species [65]. However, due to specific features of Mediterranean Sea (e.g. its enclosed nature and its oceanography, [66]) the spread of NIS can be slower than in other areas [65].

Species range shifts related to climate change can affect pattern of species richness with general increase in species richness [67] due to sea warming and with ThS responsible to foster this increase [68]. However this evidence does not match the one arising from our results, where the peak of species richness (approx. 40° latitude) is recorded beyond the latitudinal limit recorded for NIS (36°) and approximately at latitude where ThS abundance drops to zero.

MPAs also appeared to have no effects on the distribution and density of thermophilic species that showed decreasing density with increasing latitude regardless of protection. The Mediterranean Sea is currently becoming warmer both in deep [69] and surface waters [70], a trend which is reflected in the increased density of ThS [15], [16], [63]. Changes in thermal conditions have been documented to drive the reorganization of fish assemblages [15], [63]. Sea warming is expected to drive a general northward shift of fish distribution ranges in the Mediterranean Sea, not yet evident in our data, leading to the gradual replacement of cold-temperate species by thermophilic species threatening the survival of endemic temperate species [15]. Continued monitoring will be critical for tracking community shifts from invasions and warming.

In conclusion, this study provides new insights about MPAs effects at spatial scales not addressed by previous studies, especially in the Mediterranean Sea. Specifically: 1) the most relevant fish responses to protection are consistent across the Mediterranean; 2) different fish species contribute to the effects of protection, likely depending on local conditions; 3) MPAs were not found to be effective on NIS invasions and/or ThS range expansion throughout the Mediterranean Sea. These results thus provide more robust expectations about the effects of new MPAs that may be established under ongoing regional and national efforts.

## Supporting Information

Figure S1 **Mean values (± SE) of index of rugosity at three different protection levels.**
(DOC)

Table S1 **Sampling site with geographical coordinates and protection level.**
(DOC)

Table S2 **Sampled fish taxa.** Sites are numbered according to sites numbers provided in Appendix 1. PL = planktivore, DE = detritivore, CA = carnivorous, AP = apex predator, HE = herbivorous. NC = no commercial value, LC = low commercial value, C = commercial value.
(DOC)

Table S3 **Full PERMANOVA tables on square root transformed univariate data.**
(DOC)

Table S4 **Full PERMANOVA tables on square root transformed multivariate data.**
(DOC)
