# Supplementary material for: Large-Scale Assessment of Mediterranean Marine Protected Areas Effects on Fish Assemblages
Source: PLoS One. 2014 Apr 16;9(4):e91841. doi: 10.1371/journal.pone.0091841 (PMC3989174; doi:10.1371/journal.pone.0091841)

**Figure S1. Mean values (± SE) of index of rugosity at three different protection levels.** Black bars indicate fished areas (F), light gray bars indicate intermediate protected MPAs (IP) and dark gray bars indicate highly protected MPAs (HP).

The index of rugosity was calculated as described in materials and methods section of the main text.


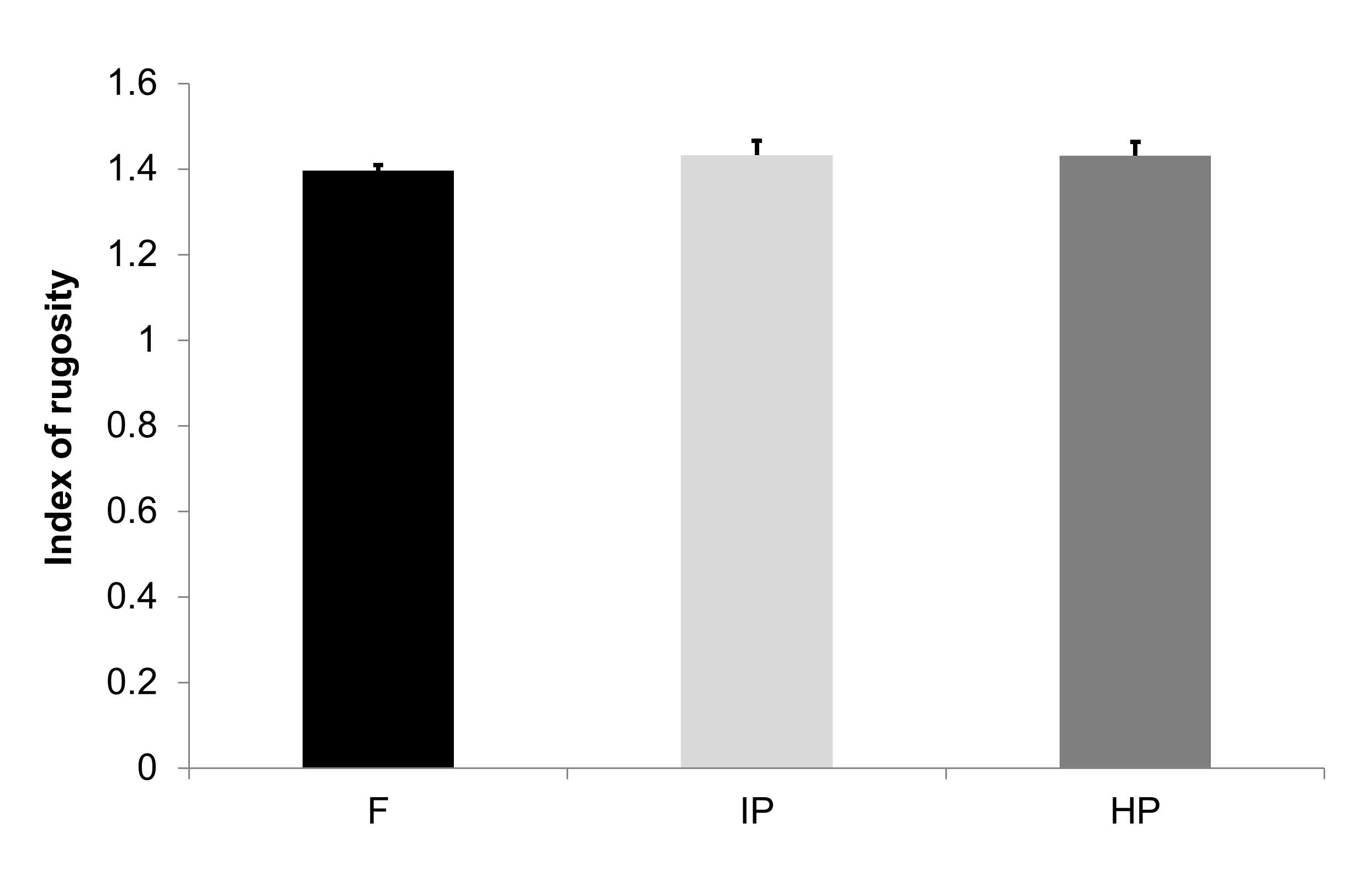

Supplement: Figure S1 — Mean values (± SE) of index of rugosity at three different protection levels. (DOC) [file pone.0091841.s001.doc]
