# Supplementary material for: Large-Scale Assessment of Mediterranean Marine Protected Areas Effects on Fish Assemblages
Source: PLoS One. 2014 Apr 16;9(4):e91841. doi: 10.1371/journal.pone.0091841 (PMC3989174; doi:10.1371/journal.pone.0091841)
Supplement: Table S1 — Sampling site with geographical coordinates and protection level. (DOC) [file pone.0091841.s002.doc]

**Table S1. Sampling site with geographical coordinates and protection level.**

| **LABEL** | **STATION** | **SITE NAME** | **LAT N** | **LONG E** | **PROTECTION REGIME** |
| --- | --- | --- | --- | --- | --- |
| 1 | ADR1 | ADRASAN | 36.2372 | 30.4358 | F |
| 1 | ADR2 | ADRASAN | 36.2561 | 30.4617 | F |
| 1 | ADR3 | ADRASAN | 36.2717 | 30.4789 | F |
| 1 | ADR4 | ADRASAN | 36.3878 | 30.4867 | F |
| 1 | ADR5 | ADRASAN | 36.3678 | 30.4867 | F |
| 1 | ADR6 | ADRASAN | 36.3369 | 30.5294 | F |
| 2 | AIR1 | ILLA DE L'AIRE | 39.8031 | 4.2948 | F |
| 2 | AIR2 | ILLA DE L'AIRE | 39.7998 | 4.2880 | F |
| 2 | AIR3 | ILLA DE L'AIRE | 39.8215 | 4.2204 | F |
| 2 | AIR4 | ILLA DE L'AIRE | 39.8212 | 4.2269 | F |
| 2 | AIR5 | ILLA DE L'AIRE | 39.8009 | 4.2817 | F |
| 2 | AIR6 | ILLA DE L'AIRE | 39.8143 | 4.2381 | F |
| 3 | ALO1 | ALONISSOS | 39.1082 | 23.7850 | IP |
| 3 | ALO2 | ALONISSOS | 39.1005 | 23.7786 | IP |
| 3 | ALO3 | ALONISSOS | 39.1519 | 23.8294 | IP |
| 3 | ALO4 | ALONISSOS | 39.1576 | 23.8407 | IP |
| 3 | ALO5 | ALONISSOS | 39.1719 | 23.8720 | IP |
| 3 | ALO6 | ALONISSOS | 39.2025 | 23.8667 | IP |
| 4 | AYV1 | AYVALIK | 39.3217 | 26.5455 | F |
| 4 | AYV2 | AYVALIK | 39.3352 | 26.5928 | F |
| 4 | AYV3 | AYVALIK | 39.3331 | 26.5683 | F |
| 4 | AYV4 | AYVALIK | 39.3356 | 26.5627 | F |
| 4 | AYV5 | AYVALIK | 39.3583 | 26.5900 | F |
| 4 | AYV6 | AYVALIK | 39.3317 | 26.5303 | F |
| 5 | CAB1 | CABRERA | 39.1263 | 2.9578 | IP |
| 5 | CAB2 | CABRERA | 39.1380 | 2.9609 | IP |
| 5 | CAB3 | CABRERA | 39.1687 | 2.9778 | IP |
| 5 | CAB4 | CABRERA | 39.2054 | 2.9781 | IP |
| 5 | CAB5 | CABRERA | 39.1295 | 2.9294 | IP |
| 5 | CAB6 | CABRERA | 39.1326 | 2.9239 | IP |
| 6 | CAP1 | CAPO CACCIA | 40.3615 | 8.0851 | IP |
| 6 | CAP2 | CAPO CACCIA | 40.3611 | 8.0835 | IP |
| 6 | CAP3 | CAPO CACCIA | 40.5690 | 8.2331 | IP |
| 6 | CAP4 | CAPO CACCIA | 40.5691 | 8.2304 | IP |
| 7 | CAR1 | CARLOFORTE | 39.1131 | 8.1845 | F |
| 7 | CAR2 | CARLOFORTE | 39.1088 | 8.1600 | F |
| 7 | CAR3 | CARLOFORTE | 39.0871 | 8.1315 | F |
| 7 | CAR4 | CARLOFORTE | 39.0848 | 8.1380 | F |
| 7 | CAR5 | CARLOFORTE | 39.0755 | 8.1467 | F |
| 7 | CAR6 | CARLOFORTE | 39.0648 | 8.1487 | F |
| 8 | CAV1 | CAVALLERIA | 40.0848 | 4.0996 | IP |
| 8 | CAV2 | CAVALLERIA | 40.0849 | 4.0955 | IP |
| 8 | CAV3 | CAVALLERIA | 40.0905 | 4.0795 | IP |
| 8 | CAV4 | CAVALLERIA | 40.0741 | 4.0941 | IP |
| 8 | CAV5 | CAVALLERIA | 40.0689 | 4.0332 | IP |
| 8 | CAV6 | CAVALLERIA | 40.0585 | 4.1249 | IP |
| 9 | CRE1 | CAP DE CREUS | 42.2453 | 3.2310 | IP |
| 9 | CRE2 | CAP DE CREUS | 42.2471 | 3.2373 | IP |
| 9 | CRE3 | CAP DE CREUS | 42.3126 | 3.3105 | IP |
| 9 | CRE4 | CAP DE CREUS | 42.2919 | 3.3075 | IP |
| 9 | CRE5 | CAP DE CREUS | 42.3325 | 3.2796 | IP |
| 9 | CRE6 | CAP DE CREUS | 42.3235 | 3.3085 | IP |
| 10 | DRA1 | DRAGONERA | 39.5888 | 2.3177 | F |
| 10 | DRA2 | DRAGONERA | 39.5985 | 2.3374 | F |
| 10 | DRA3 | DRAGONERA | 39.5860 | 2.3274 | F |
| 10 | DRA4 | DRAGONERA | 39.5741 | 2.3096 | F |
| 10 | DRA5 | DRAGONERA | 39.6097 | 2.3613 | F |
| 10 | DRA6 | DRAGONERA | 39.5632 | 2.3485 | F |
| 11 | EIV1 | EIVISSA | 38.9787 | 1.1588 | F |
| 11 | EIV2 | EIVISSA | 38.9690 | 1.1655 | F |
| 11 | EIV3 | EIVISSA | 38.9563 | 1.1889 | F |
| 11 | EIV4 | EIVISSA | 38.9598 | 1.1960 | F |
| 11 | EIV5 | EIVISSA | 38.9879 | 1.2074 | F |
| 11 | EIV6 | EIVISSA | 38.9885 | 1.2154 | F |
| 12 | FET1 | FETHIYE | 36.5572 | 29.0311 | F |
| 12 | FET2 | FETHIYE | 36.5783 | 29.0344 | F |
| 12 | FET3 | FETHIYE | 36.6036 | 29.0289 | F |
| 12 | FET4 | FETHIYE | 36.6189 | 29.0417 | F |
| 12 | FET5 | FETHIYE | 36.5961 | 29.0281 | F |
| 12 | FET6 | FETHIYE | 36.6175 | 29.0628 | F |
| 13 | FMN1 | FORMENTERA | 38.7879 | 1.4806 | HP |
| 13 | FMN2 | FORMENTERA | 38.7988 | 1.4814 | HP |
| 13 | FMN3 | FORMENTERA | 38.8032 | 1.4788 | HP |
| 13 | FMN4 | FORMENTERA | 38.7963 | 1.4800 | HP |
| 13 | FMN5 | FORMENTERA | 38.7865 | 1.4848 | HP |
| 13 | FMN6 | FORMENTERA | 38.8043 | 1.4793 | HP |
| 14 | FOR1 | FORMENTOR | 39.9563 | 3.1595 | F |
| 14 | FOR2 | FORMENTOR | 39.9460 | 3.1352 | F |
| 14 | FOR3 | FORMENTOR | 39.9585 | 3.1715 | F |
| 14 | FOR4 | FORMENTOR | 39.9559 | 3.1921 | F |
| 14 | FOR5 | FORMENTOR | 39.9429 | 3.1975 | F |
| 15 | GEN1 | GENOVA | 44.3604 | 9.1309 | F |
| 15 | GEN2 | GENOVA | 44.3622 | 9.1264 | F |
| 15 | GEN3 | GENOVA | 44.3708 | 9.0924 | F |
| 15 | GEN4 | GENOVA | 44.3670 | 9.1065 | F |
| 15 | GEN5 | GENOVA | 44.3749 | 9.0768 | F |
| 15 | GEN6 | GENOVA | 44.3781 | 9.0534 | F |
| 16 | GOK1 | GOKOVA | 37.0211 | 28.0789 | F |
| 16 | GOK2 | GOKOVA | 37.0222 | 28.1142 | F |
| 16 | GOK3 | GOKOVA | 37.0292 | 28.1611 | F |
| 16 | GOK4 | GOKOVA | 37.0308 | 28.1372 | F |
| 16 | GOK5 | GOKOVA | 37.0236 | 28.0553 | F |
| 16 | GOK6 | GOKOVA | 37.0125 | 28.1075 | F |
| 17 | GYA1 | GYAROS | 37.5833 | 24.7519 | F |
| 17 | GYA2 | GYAROS | 37.6047 | 24.6505 | F |
| 17 | GYA3 | GYAROS | 37.6001 | 24.6737 | F |
| 17 | GYA4 | GYAROS | 37.6332 | 24.7387 | F |
| 17 | GYA5 | GYAROS | 37.6287 | 24.7053 | F |
| 17 | GYA6 | GYAROS | 37.6095 | 24.6801 | F |
| 18 | KAR1 | KARPATHOS | 35.8793 | 26.8232 | F |
| 18 | KAR2 | KARPATHOS | 35.8753 | 26.8225 | F |
| 18 | KAR3 | KARPATHOS | 35.8685 | 27.2360 | F |
| 18 | KAR4 | KARPATHOS | 35.8605 | 27.2385 | F |
| 18 | KAR5 | KARPATHOS | 35.8280 | 27.2071 | F |
| 18 | KAR6 | KARPATHOS | 35.8297 | 27.2395 | F |
| 19 | KAS1 | KAS | 36.1508 | 29.6167 | F |
| 19 | KAS2 | KAS | 36.1406 | 29.6617 | F |
| 19 | KAS3 | KAS | 36.1819 | 29.6394 | F |
| 19 | KAS4 | KAS | 36.1631 | 29.6286 | F |
| 19 | KAS5 | KAS | 36.1875 | 29.6050 | F |
| 19 | KAS6 | KAS | 36.1517 | 29.6300 | F |
| 20 | KIM1 | KIMOLOS | 36.7545 | 24.6154 | F |
| 20 | KIM2 | KIMOLOS | 36.7439 | 24.6338 | F |
| 20 | KIM3 | KIMOLOS | 36.7475 | 24.6580 | F |
| 20 | KIM4 | KIMOLOS | 36.7511 | 24.6671 | F |
| 20 | KIM5 | KIMOLOS | 36.7550 | 24.5476 | F |
| 20 | KIM6 | KIMOLOS | 36.7070 | 24.5473 | F |
| 21 | MAR1 | MARATEA | 39.9322 | 15.7433 | F |
| 21 | MAR2 | MARATEA | 39.9425 | 15.7319 | F |
| 21 | MAR3 | MARATEA | 39.9908 | 15.6986 | F |
| 21 | MAR4 | MARATEA | 40.0169 | 15.6739 | F |
| 21 | MAR5 | MARATEA | 40.0378 | 15.6603 | F |
| 21 | MAR6 | MARATEA | 40.0381 | 15.6497 | F |
| 22 | MED1 | MEDES | 42.0494 | 3.2203 | HP |
| 22 | MED2 | MEDES | 42.0444 | 3.2245 | HP |
| 22 | MED3 | MEDES | 42.0414 | 3.2274 | HP |
| 22 | MED4 | MEDES | 42.0421 | 3.2253 | HP |
| 22 | MED5 | MEDES | 42.0430 | 3.2254 | HP |
| 22 | MED6 | MEDES | 42.0492 | 3.2227 | HP |
| 23 | MON1 | MONTGRI | 42.1003 | 3.1855 | F |
| 23 | MON2 | MONTGRI | 42.0959 | 3.1886 | F |
| 23 | MON3 | MONTGRI | 42.0873 | 3.1948 | F |
| 23 | MON4 | MONTGRI | 42.0845 | 3.1971 | F |
| 23 | MON5 | MONTGRI | 42.0733 | 3.2045 | F |
| 23 | MON6 | MONTGRI | 42.0668 | 3.2102 | F |
| 24 | OTR1 | OTRANTO | 40.1443 | 18.5067 | F |
| 24 | OTR2 | OTRANTO | 40.1370 | 18.5156 | F |
| 24 | OTR3 | OTRANTO | 40.1325 | 18.5168 | F |
| 24 | OTR4 | OTRANTO | 40.1107 | 18.5181 | F |
| 24 | OTR5 | OTRANTO | 40.1060 | 18.5205 | F |
| 24 | OTR6 | OTRANTO | 40.0985 | 18.5099 | F |
| 25 | PCS1 | PORTO CESAREO | 40.2473 | 17.8953 | IP |
| 25 | PCS2 | PORTO CESAREO | 40.2420 | 17.9015 | IP |
| 25 | PCS3 | PORTO CESAREO | 40.2369 | 17.9057 | IP |
| 25 | PCS4 | PORTO CESAREO | 40.2124 | 17.9183 | IP |
| 25 | PCS5 | PORTO CESAREO | 40.1980 | 17.9172 | IP |
| 25 | PCS6 | PORTO CESAREO | 40.1925 | 17.9182 | IP |
| 26 | PIP1 | PIPERI | 39.3326 | 24.3267 | F |
| 26 | PIP2 | PIPERI | 39.3449 | 24.3289 | F |
| 26 | PIP3 | PIPERI | 39.3580 | 24.3336 | F |
| 26 | PIP4 | PIPERI | 39.3699 | 24.3306 | F |
| 26 | PIP5 | PIPERI | 39.3681 | 24.3224 | F |
| 26 | PIP6 | PIPERI | 39.3672 | 24.3310 | F |
| 27 | POR1 | PORTOFINO | 44.3148 | 9.1576 | HP |
| 27 | POR2 | PORTOFINO | 44.3162 | 9.1615 | HP |
| 27 | POR3 | PORTOFINO | 44.3162 | 9.1650 | HP |
| 27 | POR4 | PORTOFINO | 44.3130 | 9.1657 | HP |
| 28 | TAV1 | TAVOLARA | 40.8761 | 9.7810 | HP |
| 28 | TAV2 | TAVOLARA | 40.8730 | 9.7784 | HP |
| 28 | TAV3 | TAVOLARA | 40.9134 | 9.7429 | HP |
| 28 | TAV4 | TAVOLARA | 40.9105 | 9.7380 | HP |
| 28 | TAV5 | TAVOLARA | 40.9021 | 9.7155 | HP |
| 29 | TGC1 | TORRE GUACETO | 40.7225 | 17.7895 | HP |
| 29 | TGC2 | TORRE GUACETO | 40.7189 | 17.7959 | HP |
| 29 | TGC3 | TORRE GUACETO | 40.7159 | 17.8057 | HP |
| 29 | TGC4 | TORRE GUACETO | 40.7189 | 17.7994 | HP |
| 29 | TGC5 | TORRE GUACETO | 40.7137 | 17.8122 | HP |
| 29 | TGC6 | TORRE GUACETO | 40.7068 | 17.8266 | HP |
| 30 | TRE1 | TREMITI | 42.2267 | 15.5439 | F |
| 30 | TRE2 | TREMITI | 42.2233 | 15.7455 | F |
| 30 | TRE3 | TREMITI | 42.2247 | 15.7514 | F |
