# Supplementary material for: Large-Scale Assessment of Mediterranean Marine Protected Areas Effects on Fish Assemblages
Source: PLoS One. 2014 Apr 16;9(4):e91841. doi: 10.1371/journal.pone.0091841 (PMC3989174; doi:10.1371/journal.pone.0091841)
Supplement: Table S2 — Sampled fish taxa. Sites are numbered according to sites numbers provided in Appendix 1. PL = planktivore, DE = detritivore, CA = carnivorous, AP = apex predator, HE = herbivorous. NC = no commercial value, LC = low commercial value, C = commercial value. (DOC) [file pone.0091841.s003.doc]

**Table S2.** Sampled fish taxa. Sites are numbered according to sites numbers provided in Appendix 1. PL= planktivore, DE= detritivore, CA= carnivorous, AP= apex predator, HE= herbivorous. NC= no commercial value, LC= low commercial value, C= commercial value.

| **TAXON** | **EXTENDED NAME** | **SITE WHERE THE TAXON WAS OBSERVED** | **COMMERCIAL INTEREST** | **TROPHIC LEVEL** |
| --- | --- | --- | --- | --- |
| ANT.ANT | *Anthias anthias* | 18 | NC | PL |
| APO.IMB | *Apogon imberbis* | 1,3,4,6,7,9,15,16,18,20,21,24-28 | NC | PL |
| ATH.SPP | *Atherina spp* | 5,16,17,19 | LC | PL |
| BOO.BOO | *Boops boops* | 4-12,14,15,18,20,22,24,27-29 | C | PL |
| CHE.LAB | *Chelon labrosus* | 9,13,22,26 | C | DE |
| CHR.CHR | *Chromis chromis* | From 1 to 30 | NC | PL |
| CLUPEI | *Clupeidae* | 8 | C | PL |
| CON.CON | *Conger conger* | 9,29 | C | AP |
| COR.JUL | *Coris julis* | From 1 to 30 | NC | CA |
| CTE.RUP | *Ctenolabrus rupestris* | 9,22,23 | LC | CA |
| DAS.PAS | *Dasyatis pastinaca* | 16,18 | LC | CA |
| DAS.SPP | *Dasyatis sp.* | 5,6,9,13,14,15,22,23,25-29 | LC | CA |
| DEN.DEN | *Dentex dentex* |  | C | AP |
| DIC.LAB | *Dicentrarchus labrax* | 9,22,27,29 | C | AP |
| DIP.ANN | *Diplodus annularis* | 2-8,10,11,13,14,15,17,20,21,23-29 | C | CA |
| DIP.CER | *Diplodus cervinus* | 9,22 | C | CA |
| DIP.PUN | *Diplodus puntazzo* | 1-6,8-10,13,15,20,22-30 | C | CA |
| DIP.SAR | *Diplodus sargus* | 1-18,20-30 | C | CA |
| DIP.VUL | *Diplodus vulgaris* | From 1 to 30 | C | CA |
| EPI.CAN | *Epinephelus caninus* | 17 | C | AP |
| EPI.COS | *Epinephelus costae* | 1,5,13,16,17,20,25,28 | C | AP |
| EPI.MAR | *Epinephelus marginatus* | 1,4,5,8,9,11-14,16,18,20-22,24,26-28 | C | AP |
| FIS.COM | *Fistularia commersonii* | 12,19 | LC | AP |
| GOB.AUR | *Gobius auratus* | 30 | NC | CA |
| GOB.BUC | *Gobius bucchichi* | 4,15,25,29,30 | NC | CA |
| GOB.COB | *Gobius cobitis* | 4 | NC | CA |
| GOB.CRU | *Gobius cruentatus* | 4,20,24 | NC | CA |
| GOB.GEN | *Gobius geniporus* | 4,12,15,19,20,24,29 | NC | CA |
| GOB.PAG | *Gobius paganellus* | 16 | NC | CA |
| GOB.VIT | *Gobius vittatus* | 6,16 | NC | CA |
| GOB.XAN | *Gobius xanthocephalus* | 4 | NC | CA |
| LAB.MER | *Labrus merula* | 2-11,13,15,17,18,20,22-29 | C | CA |
| LAB.MIX | *Labrus mixtus* | 6,22 | LC | CA |
| LAB.VIR | *Labrus viridis* | 2,4,5,7,8,10,11,13,14,18,20,22,23,28 | LC | CA |
| LIT.MOR | *Lithognathus mormyrus* | 27 | C | CA |
| LIZ.AUR | *Liza aurata* | 15 | C | DE |
| MUG.CEP | *Mugil cephalus* | 4,25 | C | DE |
| MUG.SPP | *Unidentified Mugilidae* | 27 | C | DE |
| MUL.SUR | *Mullus surmuletus* | 2,3,5-15,17-24,26,-30 | C | CA |
| MUR.HEL | *Muraena helena* | 2,6,8,13,14,15,17,25-28 | C | CA |
| MYC.RUB | *Mycteroperca rubra* | 1 | C | AP |
| OBL.MEL | *Oblada melanura* | 1-11,13-15,17-30 | C | PL |
| PAG.ACA | *Pagellus acarne* | 7 | C | CA |
| PAG.PAG | *Pagrus pagrus* | 23-25,29 | C | CA |
| PAR.GAT | *Parablennius gattorugine* | 24 | NC | CA |
| PAR.ROU | *Parablennius rouxi* | 3,6,7,24,25,27-30 | NC | CA |
| PHY.PHY | *Phycis phycis* | 4,6,9,11,27 | C | CA |
| POM.QUA | *Pomatoschistus quagga* | 29 | NC | CA |
| POM.SAL | *Pomatomus saltatrix* | 25 | C | AP |
| PTE.PEL | *Pteragogus pelycus* | 12 | NC | CA |
| SAR.RUB | *Sargocentron rubrum* | 1,12,16 | NC | CA |
| SAR.SAL | *Sarpa salpa* | 2-11,13-15,17,18,20-30 | LC | HE |
| SCI.UMB | *Sciaena umbra* | 3,5,6,13,20,22,25-28 | C | CA |
| SCO.MAD | *Scorpaena maderensis* | 16,18,20,25,26 | C | CA |
| SCO.NOT | *Scorpaena notata* | 6,15,24 | C | CA |
| SCO.POR | *Scorpaena porcus* | 7,15,21,24,25,27,29 | C | CA |
| SCO.SCR | *Scorpaena scrofa* | 6,13,22,26 | C | CA |
| SER.DUM | *Seriola dumerili* | 8,11,15 | C | AP |
| SER.CAB | *Serranus cabrilla* | 2,-10,12,14-18,20-30 | C | CA |
| SER.SCR | *Serranus scriba* | 1-21,23-25,27-30 | C | CA |
| SIG.LUR | *Siganus luridus* | 1,12,16,18-20 | LC | HE |
| SIG.RIV | *Siganus rivulatus* | 1,12,16,19 | LC | HE |
| SPA.AUR | *Sparus aurata* | 9,16,22,25,27-29 | C | CA |
| SPA.CRE | *Sparisoma cretense* | 1,12,16-21,25 | C | HE |
| SPH.VIR | *Sphyraena viridensis* | 5,8,11,13,22,28 | C | AP |
| SPI.MAE | *Spicara maena* | 6,7,9,10,13,15,18,19,21,22,25-28 | LC | PL |
| SPI.SMA | *Spicara smaris* | 4,9,12,17,18,21,25,29 | LC | PL |
| SPO.CAN | *Spondyliosoma cantharus* | 3,5-11,13-15,17,21-30 | C | CA |
| SYM.CIN | *Symphodus cinereus* | 4,15,25,29 | NC | CA |
| SYM.DOD | *Symphodus doderleini* | 3-5,7,10,13,15,16,18,20-30 | NC | CA |
| SYM.MED | *Symphodus mediterraneus* | From 2 to 30 | NC | CA |
| SYM.MEL | *Symphodus melanocercus* | 2-5,7-11,13-15,20,22,23,26-28 | NC | CA |
| SYM.OCE | *Symphodus ocellatus* | 2-11,13-15,20-30 | NC | CA |
| SYM.ROI | *Symphodus roissali* | 2,4-11,13,15,20-30 | NC | CA |
| SYM.ROS | *Symphodus rostratus* | 3-10,13,15,18,20-29 | NC | CA |
| SYM.TIN | *Symphodus tinca* | 2-11,13-30 | LC | CA |
| SYN.SAU | *Synodus saurus* | 29 | NC | AP |
| THA.PAV | *Thalassoma pavo* | 1-21,24-28,30 | NC | CA |
| TRI.DEL | *Tripterygion delaisi* | 1,4,6,7,12,15,24,25,29 | NC | CA |
| TRI.MEL | *Tripterygion melanurus* | 6 | NC | CA |
| TRI.TRI | *Tripterygion tripteronotus* | 3,4,6 | NC | CA |
| TRI.MIN | *Trisopterus minutus* | 6 | C | CA |
