# Supplementary material for: Large-Scale Assessment of Mediterranean Marine Protected Areas Effects on Fish Assemblages
Source: PLoS One. 2014 Apr 16;9(4):e91841. doi: 10.1371/journal.pone.0091841 (PMC3989174; doi:10.1371/journal.pone.0091841)
Supplement: Table S3 — Full PERMANOVA tables on square root transformed univariate data. (DOC) [file pone.0091841.s004.doc]

**Table S3.** Full PERMANOVA tables on square root transformed univariate data. For factors labels see text. Tables are provided in the same order they are referred in the main text.

**a. Total density of fish (all taxa pooled).**

| **Source** | **df** | **SS** | **MS** | **Pseudo-F** | **P(perm)** |
| --- | --- | --- | --- | --- | --- |
| Ru | 1 | 226.02 | 226.02 | 6.4239 | 0.0115 |
| Pr | 2 | 147.35 | 73.676 | 0.94722 | 0.4019 |
| SI(Pr) | 27 | 2085.5 | 77.239 | 3.9483 | 0.0001 |
| ST(SI(Pr)) | 141 | 2765.5 | 19.614 | 2.4471 | 0.0001 |
| Res | 341 | 2733.1 | 8.015 |  |  |
| Total | 512 | 7957.5 |  |  |  |

**b. Total biomass of fish (all taxa pooled).**

| **Source** | **df** | **SS** | **MS** | **Pseudo-F** | **P(perm)** |
| --- | --- | --- | --- | --- | --- |
| Ru | 1 | 58.628 | 58.628 | 7.6923 | 0.007 |
| Pr | 2 | 1325.1 | 662.56 | 40.567 | 0.0001 |
| SI(Pr) | 27 | 437.39 | 16.2 | 4.0183 | 0.0001 |
| ST(SI(Pr)) | 141 | 569.49 | 4.039 | 1.7172 | 0.0002 |
| Res | 341 | 802.04 | 2.352 |  |  |
| Total | 512 | 3192.7 |  |  |  |

**c. Species richness.**

| **Source** | **df** | **SS** | **MS** | **Pseudo-F** | **P(perm)** |
| --- | --- | --- | --- | --- | --- |
| Ru | 1 | 1.7855 | 1.7855 | 1.8149 | 0.1792 |
| Pr | 2 | 31.288 | 15.644 | 6.1535 | 0.0055 |
| SI(Pr) | 27 | 68.086 | 2.5217 | 10.655 | 0.0001 |
| ST(SI(Pr)) | 141 | 33.45 | 0.23723 | 2.1798 | 0.0001 |
| Res | 341 | 37.112 | 0.10883 |  |  |
| Total | 512 | 171.72 |  |  |  |

**d. Total density of apex predator.**

| **Source** | **df** | **SS** | **MS** | **Pseudo-F** | **P(perm)** |
| --- | --- | --- | --- | --- | --- |
| Ru | 1 | 0.93631 | 0.93631 | 2.4354 | 0.1215 |
| Pr | 2 | 26.675 | 13.337 | 25.045 | 0.0001 |
| SI(Pr) | 27 | 14.264 | 0.52829 | 1.4647 | 0.0818 |
| ST(SI(Pr)) | 141 | 50.911 | 0.36107 | 1.3295 | 0.022 |
| Res | 341 | 92.608 | 0.27158 |  |  |
| Total | 512 | 185.39 |  |  |  |

**e. Total biomass of apex predator.**

| **Source** | **df** | **SS** | **MS** | **Pseudo-F** | **P(perm)** |
| --- | --- | --- | --- | --- | --- |
| Ru | 1 | 27.337 | 27.337 | 5.9988 | 0.0132 |
| Pr | 2 | 529.45 | 264.72 | 37.331 | 0.0001 |
| SI(Pr) | 27 | 189.92 | 7.0341 | 1.606 | 0.0426 |
| ST(SI(Pr)) | 141 | 618.76 | 4.3884 | 1.7865 | 0.0004 |
| Res | 341 | 837.64 | 2.4564 |  |  |
| Total | 512 | 2203.1 |  |  |  |

**f. Total density of carnivores.**

| **Source** | **df** | **SS** | **MS** | **Pseudo-F** | **P(perm)** |
| --- | --- | --- | --- | --- | --- |
| Ru | 1 | 29.893 | 29.893 | 2.6265 | 0.113 |
| Pr | 2 | 311.61 | 155.81 | 5.3281 | 0.0101 |
| Si(Pr) | 27 | 783.17 | 29.006 | 9.7398 | 0.0001 |
| St(Si(Pr)) | 141 | 421 | 2.9858 | 2.3954 | 0.0001 |
| Res | 341 | 425.05 | 1.2465 |  |  |
| Total | 512 | 1970.7 |  |  |  |

**g. Total biomass of carnivores.**

| **Source** | **df** | **SS** | **MS** | **Pseudo-F** | **P(perm)** |
| --- | --- | --- | --- | --- | --- |
| Ru | 1 | 27.94 | 27.94 | 3.2655 | 0.0735 |
| Pr | 2 | 800.38 | 400.19 | 18.692 | 0.0001 |
| SI(Pr) | 27 | 573.36 | 21.236 | 8.492 | 0.0001 |
| ST(SI(Pr)) | 141 | 353.36 | 2.5061 | 1.9707 | 0.0001 |
| Res | 341 | 433.63 | 1.2717 |  |  |
| Total | 512 | 2188.7 |  |  |  |

**h. Total density of detritivorous.**

| **Source** | **df** | **SS** | **MS** | **Pseudo-F** | **P(perm)** |
| --- | --- | --- | --- | --- | --- |
| Ru | 1 | 3.2746E-2 | 3.2746E-2 | 0.51726 | 0.4703 |
| Pr | 2 | 0.58295 | 0.29148 | 4.8952 | 0.0172 |
| Si(Pr) | 27 | 1.5974 | 5.9164E-2 | 0.84617 | 0.6794 |
| St(Si(Pr)) | 141 | 9.8633 | 6.9953E-2 | 1.1182 | 0.1178 |
| Res | 341 | 21.333 | 6.256E-2 |  |  |
| Total | 512 | 33.409 |  |  |  |

**i. Total biomass of detritivorous.**

| **Source** | **df** | **SS** | **MS** | **Pseudo-F** | **P(perm)** |
| --- | --- | --- | --- | --- | --- |
| Ru | 1 | 1.9661E-2 | 1.9661E-2 | 4.5425E-2 | 0.8325 |
| Pr | 2 | 4.7739 | 2.387 | 6.0062 | 0.0109 |
| SI(Pr) | 27 | 10.659 | 0.39478 | 0.81927 | 0.7481 |
| ST(SI(Pr)) | 141 | 67.973 | 0.48208 | 1.1112 | 0.0906 |
| Res | 341 | 147.94 | 0.43385 |  |  |
| Total | 512 | 231.37 |  |  |  |

**j. Total density of planktivorous.**

| **Source** | **df** | **SS** | **MS** | **Pseudo-F** | **P(perm)** |
| --- | --- | --- | --- | --- | --- |
| Ru | 1 | 10.486 | 10.486 | 1.2667 | 0.2632 |
| Pr | 2 | 5.8088 | 2.9044 | 0.24833 | 0.78 |
| Si(Pr) | 27 | 318.23 | 11.786 | 1.2863 | 0.1683 |
| St(Si(Pr)) | 141 | 1294.8 | 9.1832 | 2.0234 | 0.0001 |
| Res | 341 | 1547.6 | 4.5385 |  |  |
| Total | 512 | 3177 |  |  |  |

**k. Total biomass of planktivorous.**

| **Source** | **df** | **SS** | **MS** | **Pseudo-F** | **P(perm)** |
| --- | --- | --- | --- | --- | --- |
| Ru | 1 | 1.1039 | 1.1039 | 2.2631 | 0.1291 |
| Pr | 2 | 3.6135 | 1.8068 | 2.5751 | 0.0873 |
| SI(Pr) | 27 | 18.818 | 0.69696 | 1.2799 | 0.162 |
| ST(SI(Pr)) | 141 | 76.957 | 0.5458 | 2.0823 | 0.0002 |
| Res | 341 | 89.382 | 0.26212 |  |  |
| Total | 512 | 189.87 |  |  |  |

**l. Total density of herbivorous.**

| **Source** | **df** | **SS** | **MS** | **Pseudo-F** | **P(perm)** |
| --- | --- | --- | --- | --- | --- |
| Ru | 1 | 31.613 | 31.613 | 3.806 | 0.0608 |
| Pr | 2 | 3.5887 | 1.7944 | 9.6272E-2 | 0.9096 |
| Si(Pr) | 27 | 504.94 | 18.701 | 5.351 | 0.0001 |
| St(Si(Pr)) | 141 | 493.58 | 3.5006 | 1.5695 | 0.0011 |
| Res | 341 | 760.54 | 2.2303 |  |  |
| Total | 512 | 1794.3 |  |  |  |

**m. Total biomass of herbivorous.**

| **Source** | **df** | **SS** | **MS** | **Pseudo-F** | **P(perm)** |
| --- | --- | --- | --- | --- | --- |
| Ru | 1 | 28.87 | 28.87 | 6.4424 | 0.0128 |
| Pr | 2 | 3.1888 | 1.5944 | 0.16482 | 0.8553 |
| SI(Pr) | 27 | 261.12 | 9.6711 | 4.2317 | 0.0001 |
| ST(SI(Pr)) | 141 | 322.85 | 2.2897 | 1.7491 | 0.0002 |
| Res | 341 | 446.39 | 1.3091 |  |  |
| Total | 512 | 1062.4 |  |  |  |

**n. Total density of commercially valuable fishes.**

| **Source** | **df** | **SS** | **MS** | **Pseudo-F** | **P(perm)** |
| --- | --- | --- | --- | --- | --- |
| Ru | 1 | 6.4127 | 6.4127 | 0.7907 | 0.3751 |
| Pr | 2 | 365.43 | 182.71 | 13.319 | 0.0003 |
| SI(Pr) | 27 | 367.44 | 13.609 | 1.7542 | 0.0202 |
| ST(SI(Pr)) | 141 | 1096.6 | 7.777 | 2.2746 | 0.0001 |
| Res | 341 | 1165.9 | 3.4191 |  |  |
| Total | 512 | 3001.8 |  |  |  |

**o. Total biomass of commercially valuable fishes.**

| **Source** | **df** | **SS** | **MS** | **Pseudo-F** | **P(perm)** |
| --- | --- | --- | --- | --- | --- |
| Ru | 1 | 24.566 | 24.566 | 4.6717 | 0.0333 |
| Pr | 2 | 1448.9 | 724.47 | 77.166 | 0.0001 |
| SI(Pr) | 27 | 251.43 | 9.3121 | 2.12 | 0.0022 |
| ST(SI(Pr)) | 141 | 620.73 | 4.4023 | 1.9932 | 0.0001 |
| Res | 341 | 753.14 | 2.2086 |  |  |
| Total | 512 | 3098.8 |  |  |  |

**p. Total density of low value fishes.**

| **Source** | **df** | **SS** | **MS** | **Pseudo-F** | **P(perm)** |
| --- | --- | --- | --- | --- | --- |
| Ru | 1 | 52.206 | 52.206 | 6.8359 | 0.0116 |
| Pr | 2 | 33.452 | 16.726 | 1.0548 | 0.3586 |
| SI(Pr) | 27 | 425.26 | 15.75 | 3.6065 | 0.0001 |
| ST(SI(Pr)) | 141 | 616.89 | 4.3751 | 1.7035 | 0.0002 |
| Res | 341 | 875.8 | 2.5683 |  |  |
| Total | 512 | 2003.6 |  |  |  |

**q. Total biomass of low value fishes.**

| **Source** | **df** | **SS** | **MS** | **Pseudo-F** | **P(perm)** |
| --- | --- | --- | --- | --- | --- |
| Ru | 1 | 33.669 | 33.669 | 5.3529 | 0.0195 |
| Pr | 2 | 160.86 | 80.429 | 5.5419 | 0.0097 |
| SI(Pr) | 27 | 388.72 | 14.397 | 5.5874 | 0.0001 |
| ST(SI(Pr)) | 141 | 363.98 | 2.5814 | 1.6928 | 0.0001 |
| Res | 341 | 519.99 | 1.5249 |  |  |
| Total | 512 | 1467.2 |  |  |  |

**r. Total density of fish of null commercial value.**

| **Source** | **df** | **SS** | **MS** | **Pseudo-F** | **P(perm)** |
| --- | --- | --- | --- | --- | --- |
| Ru | 1 | 183.99 | 183.99 | 4.8 | 0.0258 |
| Pr | 2 | 29.347 | 14.673 | 0.17406 | 0.8404 |
| SI(Pr) | 27 | 2272.7 | 84.175 | 4.0773 | 0.0001 |
| ST(SI(Pr)) | 141 | 2918.1 | 20.696 | 2.26 | 0.0001 |
| Res | 341 | 3122.7 | 9.1575 |  |  |
| Total | 512 | 8526.8 |  |  |  |

**s. Total biomass of fish of null commercial value.**

| **Source** | **df** | **SS** | **MS** | **Pseudo-F** | **P(perm)** |
| --- | --- | --- | --- | --- | --- |
| Ru | 1 | 0.3528 | 0.3528 | 0.12732 | 0.7201 |
| Pr | 2 | 11.236 | 5.6181 | 0.93531 | 0.4025 |
| SI(Pr) | 27 | 161.08 | 5.9659 | 4.0497 | 0.0001 |
| ST(SI(Pr)) | 141 | 208.15 | 1.4762 | 1.8885 | 0.0001 |
| Res | 341 | 266.56 | 0.78169 |  |  |
| Total | 512 | 647.37 |  |  |  |
