# Supplementary material for: Large-Scale Assessment of Mediterranean Marine Protected Areas Effects on Fish Assemblages
Source: PLoS One. 2014 Apr 16;9(4):e91841. doi: 10.1371/journal.pone.0091841 (PMC3989174; doi:10.1371/journal.pone.0091841)
Supplement: Table S4 — Full PERMANOVA tables on square root transformed multivariate data. (DOC) [file pone.0091841.s005.doc]

**Table S4.** Full PERMANOVA tables on square root transformed multivariate data. For factors labels see text. Tables are provided in the same order they are referred in the main text.

a. Multivariate density of commercially valuable fishes.

| **Source** | **df** | **SS** | **MS** | **Pseudo-F** | **P(perm)** |
| --- | --- | --- | --- | --- | --- |
| Ru | 1 | 3877.3 | 3877.3 | 0.67377 | 0.6554 |
| Pr | 2 | 48848 | 24424 | 1.7555 | 0.0681 |
| SI(Pr) | 27 | 3.7275E5 | 13806 | 6.779 | 0.0001 |
| ST(SI(Pr)) | 141 | 2.8777E5 | 2040.9 | 1.9552 | 0.0001 |
| Res | 341 | 3.5596E5 | 1043.9 |  |  |
| Total | 512 | 1.0692E6 |  |  |  |

**b. Multivariate biomass of commercially valuable fishes.**

| **Source** | **df** | **SS** | **MS** | **Pseudo-F** | **P(perm)** |
| --- | --- | --- | --- | --- | --- |
| Ru | 1 | 4037.4 | 4037.4 | 0.70465 | 0.6717 |
| Pr | 2 | 79734 | 39867 | 2.9418 | 0.0013 |
| SI(Pr) | 27 | 3.6302E5 | 13445 | 6.123 | 0.0001 |
| ST(SI(Pr)) | 141 | 3.1024E5 | 2200.3 | 1.8405 | 0.0001 |
| Res | 341 | 4.0765E5 | 1195.5 |  |  |
| Total | 512 | 1.1647E6 |  |  |  |

c. Multivariate density of low value fishes.

| **Source** | **df** | **SS** | **MS** | **Pseudo-F** | **P(perm)** |
| --- | --- | --- | --- | --- | --- |
| Ru | 1 | 11371 | 11371 | 1.8975 | 0.1144 |
| Pr | 2 | 46691 | 23345 | 1.6089 | 0.1327 |
| SI(Pr) | 27 | 3.8876E5 | 14398 | 7.1546 | 0.0001 |
| ST(SI(Pr)) | 141 | 2.8431E5 | 2016.3 | 1.7684 | 0.0001 |
| Res | 341 | 3.888E5 | 1140.2 |  |  |
| Total | 512 | 1.1199E6 |  |  |  |

**d. Multivariate biomass of low value fishes.**

| **Source** | **df** | **SS** | **MS** | **Pseudo-F** | **P(perm)** |
| --- | --- | --- | --- | --- | --- |
| Ru | 1 | 10751 | 10751 | 1.8358 | 0.1297 |
| Pr | 2 | 52628 | 26314 | 1.8485 | 0.0924 |
| SI(Pr) | 27 | 3.8137E5 | 14125 | 7.3917 | 0.0001 |
| ST(SI(Pr)) | 141 | 2.6994E5 | 1914.5 | 1.7341 | 0.0001 |
| Res | 341 | 3.7647E5 | 1104 |  |  |
| Total | 512 | 1.0912E6 |  |  |  |

e. Multivariate density of fish of null commercial value.

| **Source** | **df** | **SS** | **MS** | **Pseudo-F** | **P(perm)** |
| --- | --- | --- | --- | --- | --- |
| Ru | 1 | 5720.9 | 5720.9 | 2.1417 | 0.0929 |
| Pr | 2 | 28272 | 14136 | 2.1154 | 0.0426 |
| SI(Pr) | 27 | 1.7901E5 | 6629.8 | 8.8396 | 0.0001 |
| ST(SI(Pr)) | 141 | 1.0596E5 | 751.49 | 1.7992 | 0.0001 |
| Res | 341 | 1.4243E5 | 417.68 |  |  |
| Total | 512 | 4.6139E5 |  |  |  |

**f. Multivariate biomass of fish of null commercial value.**

| **Source** | **df** | **SS** | **MS** | **Pseudo-F** | **P(perm)** |
| --- | --- | --- | --- | --- | --- |
| Ru | 1 | 5593.1 | 5593.1 | 2.0877 | 0.0957 |
| Pr | 2 | 30127 | 15063 | 2.272 | 0.0351 |
| SI(Pr) | 27 | 1.776E5 | 6577.8 | 8.4767 | 0.0001 |
| ST(SI(Pr)) | 141 | 1.0961E5 | 777.36 | 1.6648 | 0.0001 |
| Res | 341 | 1.5923E5 | 466.94 |  |  |
| Total | 512 | 4.8215E5 |  |  |  |
